# The CIC::DUX4 oncoprotein maintains DNA integrity through direct regulation of the catalytic subunit of DNA polymerase epsilon (POLE)

**DOI:** 10.1038/s41388-025-03507-9

**Published:** 2025-08-04

**Authors:** Zeinab Kosibaty, Cuyler Luck, Ross A. Okimoto

**Affiliations:** 1 Department of Medicine, University of California, San Francisco, CA USA; 2 Helen Diller Comprehensive Cancer Center, University of California, San Francisco, CA USA; 3 Biomedical Sciences Program, University of California, San Francisco, CA USA

**Keywords:** Sarcoma, Cancer genetics

## Abstract

Transcription factor (TF) fusion oncoproteins represent cancer-specific alterations that arise from chromosomal rearrangements. Through target gene recognition, TF fusions can disseminate transcriptional responses that collectively work to drive tumorigenesis. Thus, identifying the molecular targets that operate as a disease-driving network can potentially uncover key actionable dependencies. We have taken this strategy to dissect the underlying biological mechanism by which CIC::DUX4, a fusion oncoprotein associated with dismal outcomes, drives sarcomagenesis. We and others have defined a CIC::DUX4 fusion-mediated network that dysregulates cell-cycle and DNA replication checkpoints. Specifically, CIC::DUX4-mediated *CCNE1* upregulation compromises the G1/S transition, leading to high DNA replication stress and conferring a dependence on the G2/M checkpoint kinase, WEE1. WEE1 provides a molecular brake to enable effective DNA repair prior to mitotic entry. Importantly, the mechanism by which CIC::DUX4 regulates DNA repair remains unknown. Here we show that the catalytic subunit of DNA polymerase epsilon (POLE) is essential for DNA integrity and cellular division in CIC::DUX4 sarcoma. Mechanistically, POLE loss increases DNA damage and induces p21-mediated cellular senescence to limit CIC::DUX4 tumor growth in vitro and tumor formation in vivo. Collectively, we credential POLE as a CIC::DUX4 target and further characterize a functional network through which CIC::DUX4 operates to drive tumor progression and survival.

## Introduction

CIC::DUX4 sarcoma is an aggressive subtype of undifferentiated small round cell sarcoma, characterized by an oncogenic fusion between the capicua (CIC) gene and the double homeobox 4 gene (DUX4) [1, 2]. The CIC::DUX4 fusion oncoprotein retains CIC DNA-binding specificity and converts the repressor activity associated with wild-type CIC to a potent transcriptional activator that drives tumor progression and chemoresistance [2–5]. While TF fusions represent cancer-specific alterations, the CIC::DUX4 fusion remains undruggable through direct pharmacologic targeting thus, effective therapeutic strategies to target CIC::DUX4 sarcomas remain elusive. An alternative strategy that we have taken is to intercept the molecular program controlled by CIC::DUX4 [6–8]. Prior studies have highlighted the transcriptional upregulation of cell cycle-related genes in tumors expressing the CIC::DUX4 fusion oncoprotein [6, 9]. Consistent with these findings, we identified a molecular dependence on the CCNE/CDK2 complex in CIC::DUX4 sarcoma, whereby CIC::DUX4 acts as a transcriptional activator to upregulate *CCNE1* to drive sarcoma growth and survival [6]. CIC::DUX4-mediated *CCNE1* upregulation leads to rapid G1/S transition, which enhances DNA replication stress and genomic instability [6]. Using immunohistochemistry and proteomic approaches, two independent studies recently identified increased CCNE1 protein expression in patient-derived CIC::DUX4 tumor specimens, thus demonstrating the potential translational impact of our findings [10, 11].

Recently, we found that CIC::DUX4 sarcoma cells tolerate a high replicative stress state through an increased dependence on the G2/M checkpoint kinase, WEE1, which delays mitotic entry to enable DNA repair prior to mitosis [7]. Inhibition of WEE1 leads to increased DNA damage and mitotic cell death. Thus, similar to other cancers, WEE1 expression in CIC::DUX4 sarcoma provides a molecular break that delays cell-cycle progression under high replicative stress states to enable effective DNA repair prior to mitosis [12]. These studies highlight a potential vulnerability in the cellular response to replication stress that could be exploited for therapeutic benefit. Thus, a key question remains how CIC::DUX4 sarcoma cells mechanistically repair DNA under CCNE1-mediated high replicative stress states prior to mitotic entry?

In order to address this, we leveraged a chromatin profiling study [13] to identify direct transcriptional targets of the CIC::DUX4 fusion oncoprotein. Notably, we found that CIC::DUX4 maps to a putative regulatory region and modulates the expression of the catalytic subunit of DNA polymerase epsilon (POLE), an essential enzyme involved in DNA replication and repair. Specifically, POLE synthesizes the leading strand of DNA and has 3′–5′ exonuclease activity that detects and excises mismatched base pairs [14]. Notably, using RNA-seq, we observed that *POLE* was significantly downregulated following CIC::DUX4 suppression in CIC::DUX4 fusion sarcoma cells, and gene ontology analysis further indicated that *POLE* was associated with DNA damage and repair pathways [13]. Moreover, prior studies identified *POLE* as a highly specific and significantly upregulated gene in CIC-rearranged primitive neuroectodermal tumors (PNETs) compared to other non-CIC fusion positive central nervous system PNETs [15]. For these reasons, we nominated *POLE* as a key CIC::DUX4 target gene that could potentially regulate DNA repair and replication in CIC::DUX4 sarcomas.

Since POLE expression associates with CIC-fusion expression and has an established role in DNA proofreading and replication, we aimed to investigate the role of POLE in the context of CIC::DUX4 sarcoma. Specifically, we focused on how *POLE* expression influences cell cycle dynamics, DNA damage response, and cellular division in CIC::DUX4 model systems. Understanding the regulatory mechanisms governing POLE expression exposes a key piece within the CIC::DUX4 molecular network that can be targeted to potentially improve patient outcomes.

## Materials/subjects and methods

### Cell lines and culture conditions

NCC_CDS1_X1_C1 and NCC_CDS2_C1 patient-derived cell lines were previously generated by Tadashi Kondo [16, 17]. They were grown in RPMI 1640 medium supplemented with 10% fetal bovine serum (FBS). HEK293T, NIH/3T3, and C2C12 cell lines were obtained from the American Type Culture Collection. These cell lines were grown in DMEM high-glucose medium supplemented with L-Glutamine, Sodium Pyruvate (SH30243.02, Cytiva) and 10% FBS. All cell lines were maintained at 37 °C in a humidified atmosphere with 5% CO_2_. All cell lines were routinely checked for mycoplasma.

### Western blot analysis

Protein extraction was performed using RIPA buffer supplemented with proteinase and phosphatase inhibitors (Thermo Fisher Scientific). Protein concentrations were determined using the BCA Protein Assay Kit (Thermo Fisher Scientific). Equal amounts of protein were boiled and separated by Criterion TGX 4–15% gels (Bio-Rad) and transferred to nitrocellulose membranes. The membranes were blocked in 5% BSA in TBS-T and then probed with primary antibodies against POLE (Santa Cruz Biotechnology, sc-390785, 1:1000), p21 ^Waf1/Cip1^ (12D1) (Cell Signaling Technology (CST) 2947, 1:500), p53 (CST, 9282, 1:1000), Anti-Pea3 (ETV4) (San Cruz, PEA3 Antibody [16]: sc-113, 1:500), ETV5 (CST, 16274, 1:1000), DUX4 clone P4H2 (Invitrogen MA5-16147, 1:1000), Phosph-Histon H2AX (Ser139) (CST, 2577, 1;500), Capicua CIC (Abcam, ab123822, 1:500), ACTIN (CST, 4970, 1:1000), Phospho-CHK1 ser345 (133D3) (CST,2348, 1:1000) and CHK1 (CST, 2360, 1:1000), HA-tag C29F4 (CST, 3724, 1:1000). Detection was performed using appropriate secondary antibodies HRP-linked Rabbit IgG (CST, 7074, 1:3000), and HRP-linked Mouse IgG (CST,7076, 1:3000). The proteins were visualized using SuperSignal West Femto Maximum Sensitivity Substrate (Thermo Fisher Scientific), and images were captured using a Bio-Rad ChemiDoc Touch system (Bio-Rad Laboratories). All immunoblots represent at least three independent experiments.

### RNA isolation and quantitative real-time PCR

Total RNA was extracted using the RNeasy Mini Kit (Qiagen) in accordance with the manufacturer’s suggested protocol. Complementary DNA (cDNA) was synthesized from total RNA using the SensiFAST cDNA Synthesis Kit (Bioline). Quantitative real-time (RT-PCR) was carried out with TaqMan Fast Advanced Master Mix (Applied Biosystems). Expression levels of human and mouse *CIC* (recognizes CIC::DUX4), *POLE*, *CDKN1A*, *ETV4*, and *GAPDH* were measured using TaqMan probes (Thermo Fisher) on StepOnePlus Real-Time PCR system (Applied Biosystem). The specific TaqMan probes used were human *CIC* Hs00943425_g1, mouse *CIC* Mm01173214_g1, human *POLE* Hs00923952_m1, mouse *POLE* Mm00448288_m1, human *CDKN1A* Hs00355782_m1, human *ETV4* Hs00383361_g1, mouse *ETV4* Mm00476696_m1, human *GAPDH* Hs02758991_g1, and mouse *GAPDH* Mm99999915_g1. Quantitative PCR was performed with three biological replicates per cDNA sample. The relative expression of each target gene was calculated using the 2^−ΔΔCt^ method, with *GAPDH* or *ACTIN* serving as the internal control for normalization.

### Senescence β-galactosidase assay

Senescence-associated β-galactosidase activity was assessed using a staining kit (CST, #9860). Briefly, NCC_CDS1_X1_C1 and NCC_CDS2_C1 cells were transfected with si*POLE* or siControl (siCON). After 48 h, cells were fixed with the provided fixative solution and stained using the β-galactosidase staining according to the manufacturer’s instructions. The percentage of β-galactosidase positive cells was calculated from ten randomly selected fields.

### Luciferase reporter assay

The genomic sequence within the proximal *POLE* regulatory element was cloned into the pGL4.10 luciferase vector using the KpnI and HindIII restriction sites from (NEB), following the manufacturer’s protocol. The following primers were used to generate pGL4.10-POLE construct and purchased from (Integrated and Technology IDT): DNA template of *POLE* sequencing was generated using Forward primer: GGTACCGGTACCatgaaaactgaatgagagcatatgtg, and Reverse primer: AAGCTTAAGCTTagactagaaaactgctcactgg. HEK293T cells were seeded in a 96-well plate and reverse transfected with the following constructs: PGL4.10, PGL4.10-POLE, PGL4.10 with HA-CIC::DUX4, and PGL4.10-POLE with HA-CIC::DUX4. After 48 h, the cells were lysed and luciferase activity was assessed using the Luciferase Reporter Assay System (Promega) following the manufacturer’s instructions. The luminescent signal was measured with a SpectraMax M5 plate reader (Molecular Devices).

### Mutagenesis

The mutant constructs (pGL4.10-*POLE*-mut-C7, pGL4.10-*POLE*-mut-B1, pGL4.10-*POLE*-mut-C4) in the pGL4.10-*POLE* were generated using the 5 Site-Directed Mutagenesis Kit (New England BioLabs #E0554) according to the manufacturer’s protocol. All sequences were verified through Plasmidsaurus. The following primer sets were used: (forward 5′-aagcctgtgtggagtac-3′, reverse 5′-acagagctaccattgac-3′), and (forward 5′-ctgtgtggagtactgg-3′, reverse 5′-gagctaccattgac-3′). The luciferase activity from the mutant constructs was assessed using the Luciferase Reporter Assay System, as described above.

### Cell cycle analysis

NCC_CDS1_X1_C1 and NCC_CDS2_C1 cells were trypsinized, washed with PBS, and fixed in ice-cold ethanol 30 min. Cells then were treated with RNase and stained with propidium iodide solution (Thermo Fisher Scientific) and analyzed by flow cytometer (BD Fortessa™ 3). Data were analyzed using FlowJo software.

### Cell viability

Cells were prepared in culture medium at a concentration of 60,000 cells/mL. 100 µL of the cell suspension was seeded into each well of 96-well plates and treated with siRNA targeting *POLE* or control. After 48 h, the cell viability was measured in a luminometer through a CellTiter-Glo cell viability assay according to the manufacturer’s instructions (Promega Cat. #G7571). For cell counting, cells were seeded and treated as above. After 48 h, cells were harvested using trypsinization and resuspended in an appropriate culture medium. A 10 µL aliquot of the cell suspension was mixed with an equal volume of Trypan Blue. The mixture was loaded into a hemocytometer and counted using TC20 Automated Cell counter (BIO-RAD). For crystal violet assays, 100,000 cells/mL were prepared in culture medium. 500 µL of the cell suspension was seeded per well in a 12-well plate. After 48 h of transfection with *siPOLE* or control, cells were then fixed with 4% paraformaldehyde, followed by 0.05% crystal violet staining. Data were quantified from three independent images using FIJI/ImageJ (https://github.com/fiji/fiji).

### Apoptosis assays

NCC_CDS1_X1_C1 and NCC_CDS2_C1 cells were prepared in culture medium at a concentration of 60,000 cells/mL. Subsequently, 100 µL of the cell suspension was seeded into each well of 96-well plates. The cells were then transfected with siRNA specifically targeting either *POLE* or the *CIC* (also targeting CIC::DUX4). After 48 h, Caspase-3/7 activity was measured on a Molecular Devices microplate reader using Caspase-Glo reagent according to the manufacturer’s instructions (Promega).

### Lentiviral transductions

HEK293T cells were transfected with the following plasmids: pRSV-Rev, the packaging vectors pMDG and pMDL along with either pLKO.1 P POLE-1 a gift from Richard Possemato (Addgene plasmid # 160762; http://n2t.net/addgene:160762; RRID:Addgene_160762) or pLKO.1 P POLE-2 a gift from Richard Possemato (Addgene plasmid # 160764; http://n2t.net/addgene:160764; RRID:Addgene_160764) to suppress POLE expression, or pLKO.1 GFP shRNA a gift from David Sabatini (Addgene plasmid # 30323; http://n2t.net/addgene:30323; RRID:Addgene_30323) as a control. Transfections were performed using FuGENE 6 (Promega), following the manufacturer’s protocol. For viral infection, NCC_CDS2_C1 cells were infected by adding viral supernatant, generated from the transfected HEK293T cells, directly onto the target cells in the presence of 10 µg/mL Polybrene (Millipore Sigma). The viral supernatant was incubated on the cells for 48 h. Following infection, cells were maintained in culture medium supplemented with puromycin (Thermo Fisher).

### RNA sequencing and analysis

NCC_CDS1_X1_C1 cells were transfected with *siPOLE* or *siCON*. After 72 h, RNA was extracted using the RNeasy Mini kit (Qiagen) and analyzed for integrity with a TapeStation (Agilent). Samples were submitted to Novogene for library preparation and sequencing. PolyA-enriched unstranded libraries were prepared using the NEB Next Ultra II RNA Library Prep for Illumina kit. Libraries were PE150 sequenced on a Novaseq 6000 (Illumina). Briefly, FASTQ files were aligned to the human GRCh38 genome with STAR, including quantitation of read counts using the argument --quantMode GeneCounts. Uniquely mapped read rates were between 90 and 94% for all samples. A custom R script (version 4.2.2) [18, 19] was used to perform differential gene expression analysis. Briefly, column 2 from the STAR GeneCount output was merged across all samples. Then, edgeR (version 3.40.2) [20–23] was used for differential expression analysis using a GLM approach to process all samples together and quasi-likelihood exact test were used for comparing control and *siPOLE* conditions within each cell line. Resulting *p*-values were FDR-adjusted. Log2(counts per million) values were extracted from the TMM-normalized dataset using the cpm function. Code for initial processing and edgeR analysis of the data are available at https://github.com/cuylerluck/ZK_CICDUX4_POLE.

### Functional enrichment analysis

Kyoto Encyclopedia of Genes and Genomes (KEGG) pathway analysis was conducted on 816 genes from NCC_CDS1_X1_C1 using the Database for Annotation, Visualization, and Integrated Discovery (DAVID) (https://david.ncifcrf.gov). The top five signaling pathways were selected with *p* < 0.001 and FDR *q* < 0.05.

### Analysis of DNA damage by comet assay

The assay was preformed using reagents from (Enzo Life Sciences). Briefly, NCC_CDS1_X1_C1 and NCC_CDS2_C1 cells were embedded in agarose on a microscope slide and subjected to electrophoresis with TBE buffer in accordance with the manufacturer’s instructions. After staining with SYBR Green, comets were visualized under a confocal microscope Leica SP5. OpenComet, a plugin for the image processing program ImageJ, was used for quantification. All images were analyzed using the comet-finding settings with background correction applied and auto head finding. An output spreadsheet generated by OpenComet contains data on DNA damage. Tail DNA (%) represents the tail DNA content as a percentage of the total comet DNA content [24].

### Knockdown and overexpression assays

ON-TARGET plus Non-Targeting Pool (# D-001810-10-20), *CIC* siRNA (#L-015185-01-0005), *POLE* siRNA (#L-020132-00-0010) were obtained from GE Dharmacon. *DUX4* siRNA (Predesigned siRNA-DUX4_Human_Ref Seq ID: NM_033178, SASI_Hs02_00360947) was obtained from Millipore Sigma.

The siRNA pools were obtained from GE Dharmacon and the sequences as follows: Non-targeting (# D-001810-10-20): UGGUUUACAUGUCGACUAA, UGGUUUACAUGUUGUGUGA, UGGUUUACAUGUUUUCUGA, UGGUUUACAUGUUUUCCUA. Human *siPOLE* (#L-020132-00-0010): GCGAGGAACAGGCGAAAUA, GGAGGAGGGUGCUUCGUAU, GGACAGGCGUUACGAGUUC, CUCGGAAGCUGGAAGAUUA. Human *siCIC* (#L-015185-01-0005): GCUUAGUGUAUUCGGACAA, CGGCGCAAGAGACCCGAAA, GAGAAGCCGCAAUGAGCGA, CGAGUGAUGAGGAGCGCAU.

Transfections were performed in a well of a 6-well plate using siRNAs with OptiMEM (Gibco) and Lipofectamine RNAiMAX (Thermo Fisher) for 48 or 72 h. For overexpression experiments, pcDNA3.1-HA-CIC::DUX4 was a gift from Takuro Nakamura [2], and pMYs-CIC::DUX4-IRES-EGFP was a gift from Michael Kyba. The empty vector (EV) pMY-IRES-eGFP was purchased from Addgene (163361). Plasmids were transfected using OptiMEM (Gibco) and FuGENE 6 (Promega) for 48 h.

### In vivo tumor xenografts

Five to six-week-old female nude mice (NU/J) were obtained from Jackson Laboratory. All procedures were conducted under specific pathogen-free conditions and approved by the American Association for Accreditation of Laboratory Animal Care. For subcutaneous injections, NCC_CDS2_C1 cells expressing sh*POLE_1*, sh*POLE_2*, or shGFP were resuspended in a 50% PBS/50% Matrigel matrix (Corning 356234) to achieve a concentration of 420,000 cells per 50 µL injection. Mice were closely monitored for tumor formation and tumor volume was calculated using 3 independent measurements (length*width*height). A tumor was defined as ≥50 mm^3^. The tumor volume curve represents the average tumor volumes at the indicated time point for the different mouse cohorts, sh*POLE_1*, sh*POLE_2*, and shGFP. For protein Western Blot (WB) analysis, tumor xenografts were collected and lysed in RIPA buffer supplemented with protease and phosphatase inhibitors. The lysates were then homogenized using a Precellys Evolution tissue homogenizer (Bertin Technologies) with high-impact zirconium beads (Benchmark Scientific, D1032-30) to disrupt the tissues thoroughly. Following homogenization, the samples were subjected to sonication and then centrifuged at 14,000 × *g* for 15 min at 4 °C. Western blotting was performed as described above.

### Statistical analysis

Experimental data are presented as mean ± standard deviation or standard error of the Mean. *P* values for all in vitro and in vivo experiments were determined using a two-tailed Student’s *t* test or one-way analysis of variance (ANOVA), as appropriate. A *P* value of less than 0.05 was regarded as statistically significant. GraphPad Prism (version 10.2.3) was used for data and statistical analysis. Schematic Model in Fig. 4C was created in BioRender.com.

### Study approval for animal studies

Tumor transplantation and animal surgical procedures were reviewed and approved by the UCSF IACUC, protocol AN194620-01E. For animal studies using mice, we used an *N* = 6 to detect a difference in tumor formation between shControl vs sh*POLE* groups. The selection of 6 mice per group was initially selected to detect a difference in tumor with a *P* < 0.05 using 1-way ANOVA between the groups of mice. One mouse was excluded due to hemorrhage at the injection site. Mice were not subjected to treatment once tumors were implanted and therefore, no randomization was used in this study. Cages were labeled with designated groups, including *shGFP*, *shPOLE1*, *shPOLE2*.

### Ethics approval and consent to participate

All methods in this manuscript were performed in accordance with the relevant guidelines and regulations from the UCSF Institutional Biosafety Committee (#BU178659-03). The animal studies in this manuscript were performed in accordance with the relevant guidelines and regulations from the UCSF Institutional Animal Care Use Committee, protocol #AN194620-01E. No human subjects were used in this study.

## Results

### *POLE* is a transcriptional target of the CIC::DUX4 fusion oncoprotein

**Fig. 1:**
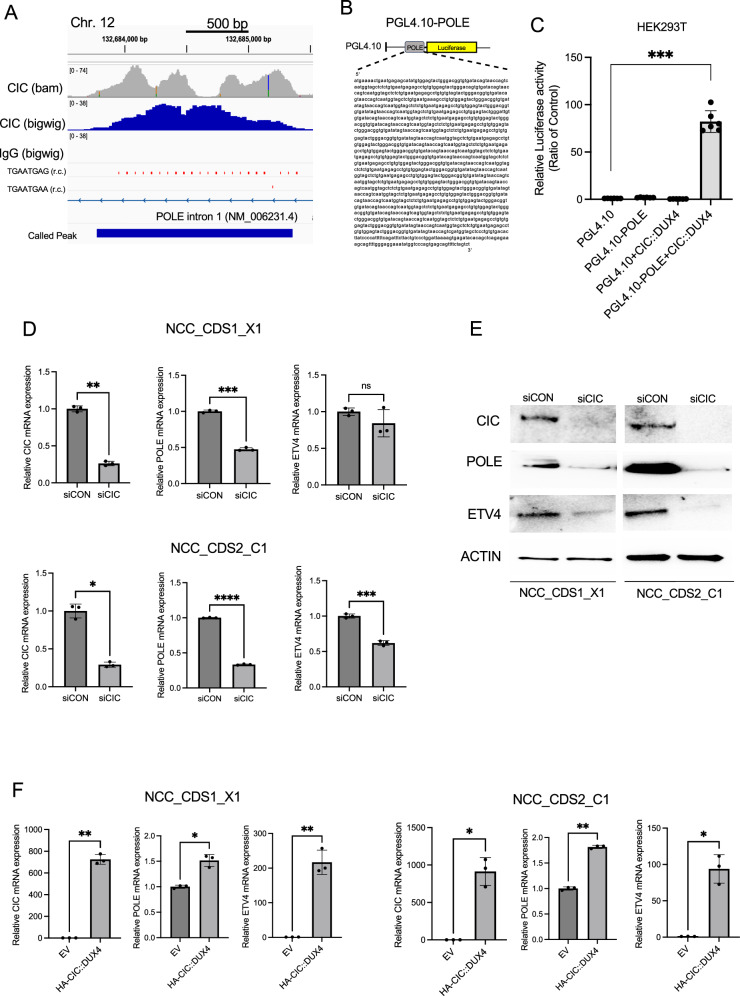
Identification of *POLE* as a transcriptional target of the CIC::DUX4 fusion oncoprotein. **A** ChIP-seq track from NCC_CDS1_X1_C1 demonstrating CIC::DUX4 occupancy (upper gray and blue) on intron 1 of *POLE*. The identified peak within the intronic region of *POLE* on chromosome 12 containing the canonical CIC::DNA binding motif TGAATGAA and multiple variant TGAATGAG motifs. Immunoglobulin (IgG) served as control. **B** Schematic of the luciferase reporter construct, PGL4.10-POLE that contains the genomic sequence of *POLE* that is occupied by CIC::DUX4. **C** Relative luciferase activity in HEK293T expressing PGL4.10, PGL4.10-POLE, PGL4.10 + CIC::DUX4, and PGL4.10-POLE + CIC::DUX4 constructs. **D** Relative mRNA expression levels of *CIC*, *POLE*, and *ETV4* in NCC_CDS1_X1_C1 (top panels) and NCC_CDS2_C1 (bottom panels) cells transfected with *siCIC* (CIC::DUX4 knockdown) or scramble control (siCON). **E** Immunoblots of CIC, POLE and ETV4 from NCC_CDS1_X1_C1 and NCC_CDS2_C1 cells transfected with *siCIC* or siCON for 48 h. **F** Relative mRNA expression levels of *CIC, POLE*, and *ETV4* in NCC_CDS1_X1_C1 (left panel) and NCC_CDS2_C1 (right panel) cells over-expressing CIC::DUX4-HA or empty vector (EV) control. Error bars represent standard deviation (SD); statistical significance was assessed Student’s *t* test. *P < 0.05, **P < 0.01, ***P < 0.001, ****P < 0.0001. Data represent results from three independent experiments.

Using our published chromatin profiling studies (Chromatin immunoprecipitation followed by sequencing, ChIP-Seq) from patient-derived CIC::DUX4 fusion positive sarcoma cells (NCC_CDS1_X1_C1), we identified 392 high-confidence targets (FDR *q* < 0.001) that overlap with CIC binding peaks [13]. Rationally informed by our prior studies that demonstrated that CIC::DUX4-mediated *CCNE1* upregulation can accelerate the G1/S transition we focused on target genes that directly regulate DNA replication and repair during S-phase. Among these genes, we identified a significant peak on chromosome 12 within the first intron of the catalytic subunit of DNA *POLE* gene. Notably, this region harbors the canonical WT-CIC DNA-binding motif, TGAATGAA, but also contained multiple (*N* = 22) variant TGAATGAG repeats that associated with a broad CIC::DUX4 binding peak (Fig. 1A). To assess whether CIC::DUX4 transcriptionally regulates *POLE* expression, we engineered pGL4.10 luciferase-based reporter constructs that contained the corresponding region that mapped to this CIC::DUX4 binding peak (pGL4.10-POLE) (Fig. 1B). Our findings reveal that ectopic expression of CIC::DUX4 increases *POLE* reporter activity through this newly identified CIC::DUX4 regulatory element compared to cells transfected with either CIC::DUX4, POLE reporter, or EV alone in HEK293T cells (Fig. 1C).

To further investigate the region required for CIC::DUX4-mediated transcriptional activity of *POLE*, we performed mutagenesis experiments to generate variants of the putative *POLE* regulatory element. Specifically, using our established pGL4.10-POLE reporter construct, we engineered the following variants: pGL4.10-POLE-mut-C7 contains 17 TGAATGAG repeats, but the consensus TGAATGAA motif is deleted; pGL4.10-POLE-mut-B1 contains six TGAATGAG repeats and retains the consensus TGAATGAA sequence; and pGL4.10-POLE-mut-C4 contains two TGAATGAG repeats (Supplementary Fig. 1A). We observed a significant decrease in luciferase activity with CIC::DUX4 co-expression in cells that harbored the pGL4.10-POLE-mut-C4 construct. In contrast, ectopic expression of CIC::DUX4 mildly reduced luciferase activity from the pGL4.10-POLE-mut-B1 construct, while pGL4.10-POLE-mut-C7 remained largely unaffected (Supplementary Fig. 1B). These findings suggest that: (1) the consensus TGAATGAA motif is not required for *POLE* expression; (2) the entire repeat-rich region is important for full CIC::DUX4-mediated transcriptional activation of *POLE* and; (3) the number to TGAATGAG sequences are positively correlated with *POLE* reporter activity.

To further validate the regulatory role of CIC::DUX4 on *POLE* expression, we performed RNA interference (RNAi) to silence endogenous *CIC::DUX4* in NCC_CDS1_X1_C1 and NCC_CDS2_C1 cells [16, 17]. Genetic silencing of *CIC::DUX4* using *siCIC* reduced POLE expression at both the mRNA and protein levels compared to siCON (ETV4 is a validated CIC::DUX4 target gene and control) (Fig. 1D, E). In order to mitigate concerns for off-target effects from *siCIC*, we knocked down CIC::DUX4 using a siRNA directed at the C-terminal end of DUX4 (retained in the fusion) in NCC_CDS1_X1_C1 and NCC_CDS2_C1 cells. Consistent with *siCIC*, we observed that genetic silencing of *DUX4* decreased POLE expression at both the mRNA and protein levels compared to siCON (Supplementary Fig. 2). Conversely, overexpression of CIC::DUX4 in these same cell lines increased *POLE* mRNA levels compared to cells transfected with an EV control (Fig. 1F). WB analysis revealed a modest increase in POLE expression in NCC_CDS2_C1 cells following CIC::DUX4 overexpression, while no qualitative change in POLE expression was detected in NCC_CDS1_X1_C1 cells (Supplementary Fig. 3). Interestingly, when CIC::DUX4 was expressed in non-sarcoma cell lines (HEK293T, C2C12, and NIH-3T3) (Supplementary Fig. 4), POLE expression remained unaffected, suggesting a cell-context specific response. Altogether, these findings suggest that CIC::DUX4 expression is necessary for *POLE* expression in cells expressing endogenous CIC::DUX4. While we observed increased *POLE* mRNA expression we did not consistently find increases in POLE protein in response to CIC::DUX4 expression. We therefore speculate that there may be a threshold level of POLE protein expression that these cells can effectively tolerate. Consistent with this, we were unable to effectively increase POLE expression upon exogenous *POLE* expression in NCC_CDS1_X1_C1 and NCC_CDS2_C1 cells in the presence or absence of CIC::DUX4 expression (data not shown).

### POLE suppression leads to cell-cycle arrest, increases DNA damage, and impairs cellular division in CIC::DUX4 sarcoma cells

**Fig. 2:**
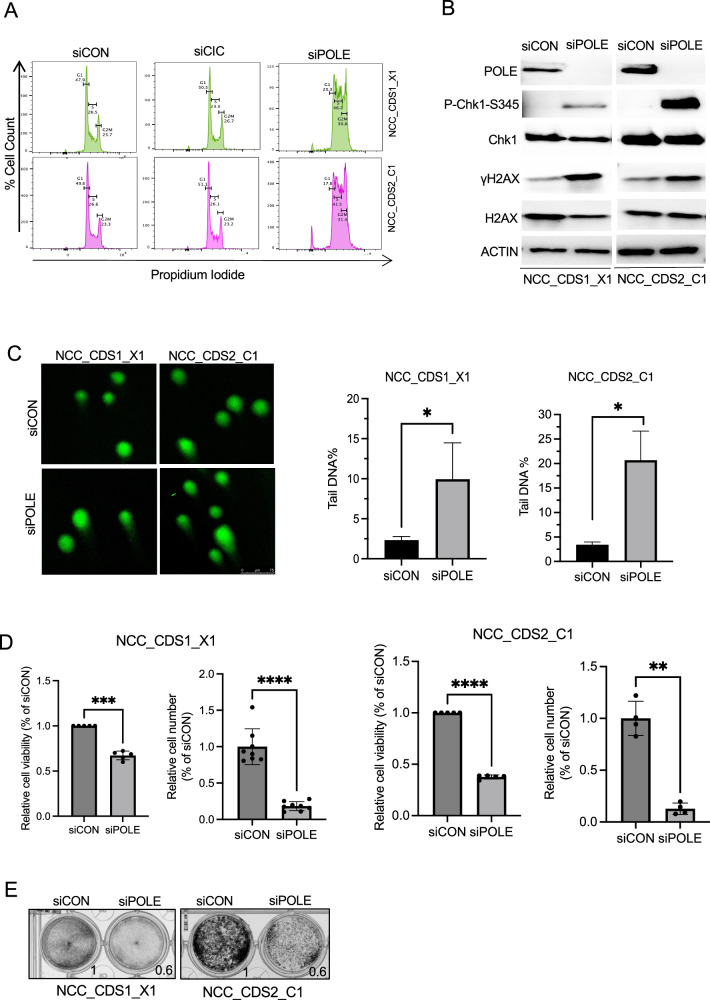
POLE suppression induces cell cycle defects, increases DNA Damage, and limits cell viability in CIC::DUX4 sarcoma cells. **A** Representative cell cycle histograms of NCC_CDS1_X1_C1 and NCC_CDS2_C1 cells 48 h post-transfection with *siPOLE*, *siCIC*, or scrambled control (siCON). Cells were stained with propidium iodide (PI) to assess DNA content. The percentage of cells in each phase of the cell cycle (G1, S, and G2/M) is indicated for each condition. **B** Immunoblots of POLE, p-Chk1 at serine 345, total Chk1, γH2AX, and total H2AX in NCC_CDS1_X1_C1 and NCC_CDS2_C1 cells expressing *siPOLE* or siCON. ACTIN was used as a loading control. Data represent results from at least three independent experiments. **C** Representative comet assay images of NCC_CDS1_X1_C1 and NCC_CDS2_C1 cells transfected with siCON or *siPOLE* for 48 h. Scale bar = 75 μm. Percentage of tail DNA contents were analyzed by OpenComet program of ImageJ software and data were presented as mean ± SEM. Comparisons between siCON and *siPOLE* were conducted using an unpaired t-test and at least 17 cells were analyzed per group from seven randomly selected images for each group. **D** Relative cell viability assays (CellTiter-Glo) and cell counts (trypan blue) of NCC_CDS1_X1_C1 and NCC_CDS2_C1 cells expressing *siPOLE* or siCON. **E** Crystal violet assay of NCC_CDS1_X1_C1 and NCC_CDS2_C1 cells expressing *siPOLE* compared with siCON. Data represent results from at least four independent experiments. *P < 0.05, **P < 0.01, ***P < 0.001, ****P < 0.0001.

POLE plays a key role in DNA replication and repair, as well as in the maintenance of genome stability [14]. Additionally, POLE has been shown to be involved in DNA synthesis, particularly during the late S phase [14]. To investigate the functional role of POLE in CIC::DUX4 sarcoma, we first examined the effect of POLE suppression on cell-cycle progression in patient-derived CIC::DUX4 sarcoma cells. Using RNAi to silence *POLE* (*siPOLE*) in NCC_CDS1_X1_C1 and NCC_CDS2_C1 cells, we observed that POLE suppression led to an increase in the population of cells in S phase, G2, as well as the sub-G1 fraction (Fig. 2A). These findings suggested that POLE loss could induce G2/M arrest to enable DNA-repair. Given these observed cell cycle effects upon POLE suppression, we hypothesized that POLE can, in part, maintain genome stability in CIC::DUX4 sarcoma cells. To explore the role of POLE on limiting DNA-damage, we performed WB analysis using validated DNA-damage markers including phosphorylation of serine 139 on the histone variant γH2AX and serine 345 on CHK1, both sensitive markers of DNA damage [25, 26] in NCC_CDS1_X1_C1 and NCC_CDS2_C1 cells ±POLE expression (Fig. 2B). Silencing POLE increased γH2AX and CHK1 phosphorylation relative to control, thus, POLE safeguards against DNA-damage in CIC::DUX4 sarcoma cells. Using an orthogonal approach to augment our findings we next conducted conventional comet assays, which are commonly used to detect and quantify DNA damage at single-cell resolution [27]. The results revealed increased DNA damage and defective repair as measured by tail DNA percentage upon POLE suppression in NCC_CDS1_X1_C1 and NCC_CDS2_C1 cells compared to control (Fig. 2C).

Next, we aimed to determine whether POLE was essential for the survival of CIC::DUX4 sarcoma cells. Using genetic silencing of *POLE* in CIC::DUX4 sarcoma cell lines (Supplementary Fig. 5), we observed that POLE suppression significantly reduced the growth and/or viability of both NCC_CDS1_X1_C1 and NCC_CDS2_C1 cells, as assessed by Cell-Titer-Glo, trypan blue staining, and crystal violet assays (Fig. 2D, E). Notably, others have characterized an induced dependence on POLE in CCNE/CDK2 activated cancers [28], thus indicating that CIC::DUX4 may operate through a similar molecular pathway. Consistent with these prior findings, we did not observe a significant impact on the viability of MCF7 breast cancer cells (known low CDK2 activity) upon POLE suppression (Supplementary Fig. 6A, B). Moreover, POLE suppression did not affect DNA-damage responses of MCF7 cells as measured by γH2AX expression (Supplementary Fig. 6C). In order to assess the relative selectivity of POLE inhibition in CIC::DUX4 sarcomas, we tested the impact of POLE expression on non-CIC::DXU4 sarcoma subtypes, including rhabdomyosarcoma cells (Rh30, RD) and Ewing sarcoma (CHLA10, A673). While POLE suppression had a relatively modest effect on rhabdomyosarcoma viability, we noted a more dramatic effect in A673 (Ewing sarcoma cells with known high replicative stress) that was not shared with other Ewing sarcoma cells (CHLA10) (Supplementary Fig. 7A, B). These findings suggest that the effects on viability that we observed are unlikely related to general toxicity from POLE suppression. Consistent with this, a recent study revealed that *POLE* was specifically upregulated in CIC::DUX4 patient-derived cells compared to other round cell sarcomas, including EWSR1::FLI1, EWSR1::ERG, and BCOR-rearrangements [29]. To further evaluate the therapeutic specificity of targeting POLE in CIC::DUX4 sarcoma cells, we treated NCC_CDS1_X1_C1 and NCC_CDS2_C1 with Aphidicolin, a DNA polymerase inhibitor (no POLE specific inhibitors are commercially available to our knowledge). This treatment resulted in a reduction in cell growth and increased DNA damage as assessed by increased phosphorylation of serine 345 on CHK1 in the treated cells compared to the control (Supplementary Fig. 8A, B).

To examine the potential role of POLE in inducing apoptosis in CIC::DUX4 sarcoma cells, we silenced POLE or CIC::DUX4 and measured Caspase 3/7 activity. Our results indicated that POLE suppression did not induce apoptosis compared to control cells (Supplementary Fig. 9). However, suppression of CIC::DUX4 resulted in an increase in Caspase 3/7 activity in the NCC_CDS1_X1_C1 and NCC_CDS2_C1 cell lines consistent with dependence on the CIC::DUX4 oncoprotein (Supplementary Fig. 9). These findings suggest that the decreased cell number observed upon POLE silencing in CIC::DUX4 sarcomas may not be operating through apoptotic pathways.

### POLE induces a senescence-like phenotype in CIC::DUX4 sarcoma cells

**Fig. 3:**
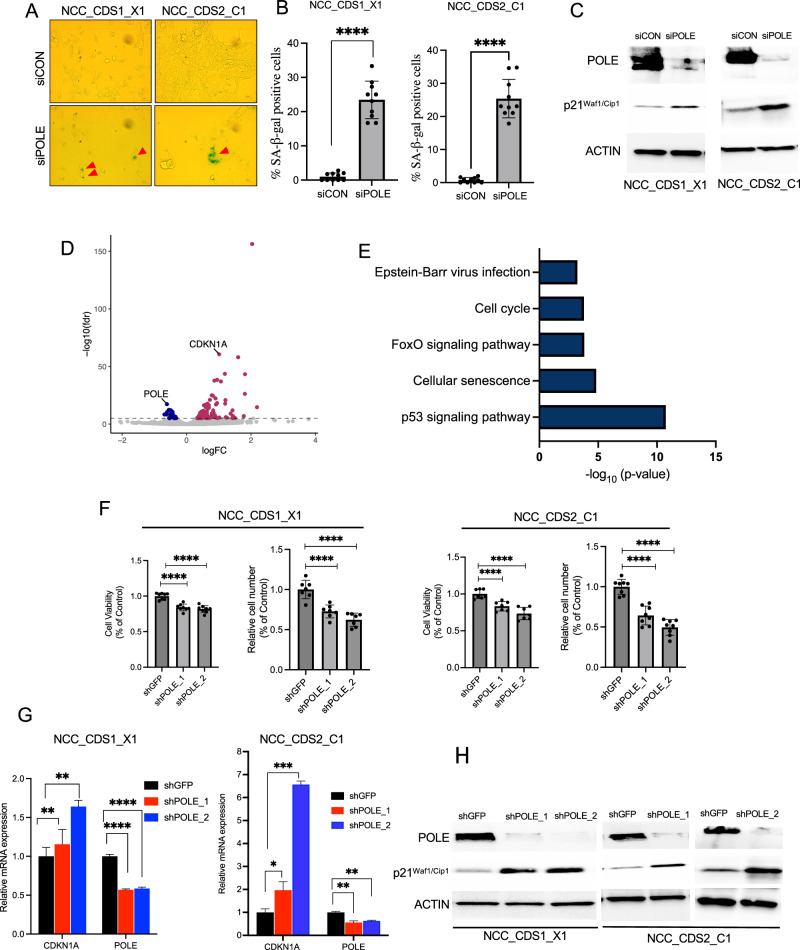
POLE Suppression induces senescence pathways in CIC::DUX4 sarcoma cells. **A** Representative images of β-galactosidase staining in NCC_CDS1_X1_C1 and NCC_CDS2_C1 cells, red arrows indicate senescent cells, which are stained green. Scale bar 100 µm. **B** Quantitative analysis of senescence-associated β-galactosidase activity in NCC_CDS1_X1_C1 and NCC_CDS2_C1 cells following *POLE* silencing (*siPOLE*) compared to control (siCON), at least 150 cells were analyzed per group from ten randomly selected images for each group. **C** Immunoblot analysis showing increased expression of p21 in NCC_CDS1_X1_C1 and NCC_CDS2_C1 cells expressing *siPOLE* compared to controls. **D** Volcano plot illustrating differentially expressed genes in NCC_CDS1_X1_C1 cells after POLE suppression, with significant genes (FDR *q* < 0.00001) highlighted; upregulated genes are indicated in red, and downregulated genes in blue. **E** KEGG pathway analysis was performed on the 816 genes from NCC_CDS1_X1_C1, with significant expression (FDR *q* < 0.01). The top five signaling pathways were highlighted with a *p* < 0.001 and FDR *q* < 0.05. **F** After 4 days of seeding, relative cell viability and cell counts were assessed in CIC::DUX4 cells stably expressing *shPOLE_1* and *shPOLE_2* compared to shGFP controls. **G**, **H** RT-PCR and immunoblot analyses of p21 (*CDKN1A*) expression in NCC_CDS2_C1 cells expressing *shPOLE_1* and *shPOLE_2* versus shGFP controls. Error bars represent mean ± SD; statistical significance was assessed using Student’s *t* test or 1-way ANOVA. *P < 0.05, **P < 0.01, ***P < 0.001, ****P < 0.0001. Data represent results from at least three independent experiments.

Our data suggested that POLE suppression does not induce cellular death through conventional apoptotic pathways in CIC::DUX4 sarcoma cells, prompting us to investigate alternative mechanisms that limited cellular division and growth. Based on our cell-cycle and biochemical analyses that were consistent with G2/M arrest due to extensive DNA-damage in POLE deficient CIC::DUX4 cells we hypothesized that the growth limiting effect is potentially mediated through cellular senescence. To test this, we performed β-galactosidase staining and biochemically assessed known markers of senescence, including p21 [30–32]. Quantitative analyses revealed a marked increase in senescence-associated β-galactosidase activity in POLE-suppressed cells compared to control cells in both NCC_CDS1_X1_C1 and NCC_CDS2_C1 cell lines (Fig. 3A, B). Additionally, western blot analysis showed increased activity of the key senescence marker p21 (Fig. 3C).

To more broadly understand how POLE regulates cellular senescence in CIC::DUX4 sarcoma cells, we conducted transcriptional profiling (RNA-Seq) in POLE-deficient and POLE-replete NCC_CDS1_X1_C1 cells. We identified differentially expressed genes (FDR *q* < 0.00001) in POLE-suppressed NCC_CDS1_X1_C1 cells (Fig. 3D and Supplementary Table 1). Consistent with our functional findings, this analysis identified differential expression of senescence-associated and DNA-damage/repair genes upon POLE suppression. Notably, genes including *CDKN1A* (gene that encodes p21) and SESN3 (Sestrin3, p53 target gene) were significantly upregulated, reinforcing the link between POLE suppression and the induction of senescence and DNA damage (Fig. 3D and Supplementary Table 1, and Supplementary Fig. 10A). In order to broaden our findings, we next suppressed *POLE* in NCC_CDS2_C1 cells and again observed an increase in *CDKN1A* and *SESN3* expression (Supplementary Fig. 10B). KEGG (Kyoto Encyclopedia of Genes and Genomes) pathway analysis of 816 differentially expressed genes with (FDR *q* < 0.01) further revealed enrichment of cellular senescence pathways, particularly the p53 signaling pathway, implicating POLE in modulating senescence through these established mechanisms (Fig. 3E and Supplementary Fig. 11). Since p53 can induce senescence through p21 we wondered if the senescence response upon POLE suppression was mediated through the p53-p21 pathway. To test this, we first silenced *POLE* and did not observe a direct effect on p53 protein levels (Supplementary Fig. 12A); but we did note p53 and p21 induction upon Nutlin-3 (inhibitor of the MDM2-p53 interaction) treatment in NCC_CDS1_X1_C1 and NCC_CDS2_C1 cells. Specifically, WB analysis revealed that Nutlin-3 treatment increased both p53 and p21 expression in both cell lines compared to DMSO-treated controls (Supplementary Fig. 12B). These findings suggest that the p53 pathway is functionally intact in CIC::DUX4 cells and regulates p21 to potentially modulate cell-cycle progression, cellular senescence, and DNA-repair mechanisms in CIC::DUX4 cells.

In order to further mitigate potential off-target effects, we established stable CIC::DUX4 cell lines expressing *shPOLE_1*, *shPOLE_2*, or *shGFP* as a control. We found that CIC::DUX4 cells expressing *shPOLE_1* and *shPOLE_2* exhibited a significant reduction in cell viability compared to control (*shGFP*) in both NCC_CDS1_X1_C1 and NCC_CDS2_C1 cell lines (Fig. 3F). RT-PCR and western blot analysis further supported our prior observations with an increase in p21 expression in NCC_CDS1_X1_C1 and NCC_CDS2_C1 cells expressing *shPOLE_1* and *shPOLE_2* compared to the control (Fig. 3G, H). These results collectively suggest that POLE plays a pivotal role in modulating cellular responses to replication stress and DNA damage, primarily through the activation of the p21-mediated senescence pathway in CIC::DUX4 sarcoma cells.

### POLE suppression inhibits tumor formation and limits growth of CIC::DUX4 sarcomas in vivo

**Fig. 4:**
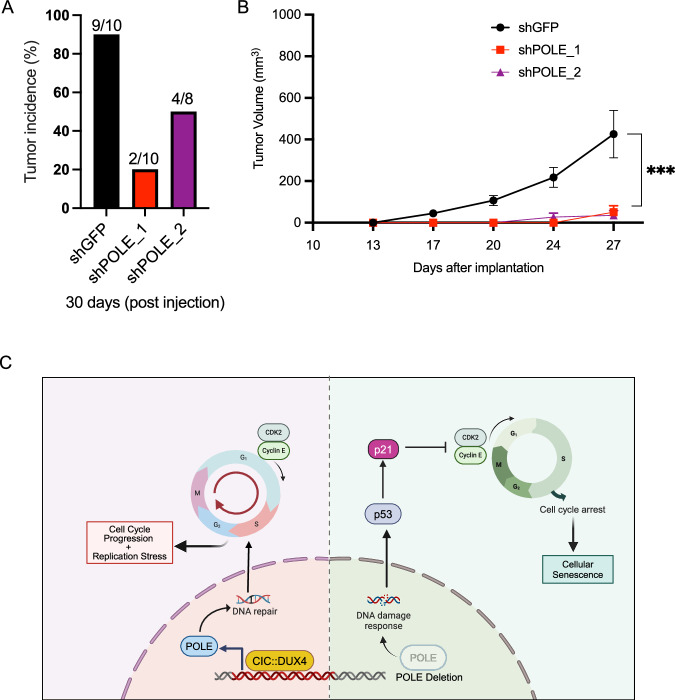
POLE suppression inhibits CIC::DUX4 tumor formation in vivo. **A** Bar graph comparing the incidence of tumor formation in mice harboring NCC_CDS2_C1 cells expressing shGFP (9/10, 90%), shPOLE_1 (2/10, 20%), or shPOLE_2 (4/8, 50%). **B** Subcutaneously implanted NCC_CDS2_C1 cells expressing shGFP (*n* = 10), shPOLE_1 (*n* = 10), or shPOLE_2 (*n* = 8). *P* value, 1-way ANOVA. Error bars represent mean ± SEM. **C** Schematic Model: CIC::DUX4-mediated regulation of POLE and its implications in cell cycle progression and DNA repair (left panel). The CIC::DUX4 fusion oncoprotein drives the transcriptional upregulation of POLE, facilitating cell cycle progression and DNA replication, and repair. Conversely, suppression of POLE promotes cellular senescence by triggering the DNA damage response and activating of p21 signaling, a native inhibitor of p21 (right). **C** was created with BioRender.com with permission.

Our in vitro experiments indicate that POLE suppression blocks tumor growth and cellular division in NCC_CDS1_X1_C1 and NCC_CDS2_C1 cells. To further investigate its effects on tumor growth in vivo, we subcutaneously implanted NCC_CDS2_C1 cells expressing *shGFP*, *shPOLE_1*, or *shPOLE_2* into the flanks of immunodeficient nude (Foxn1^nu^) mice. As expected, we found NCC_CDS2_C1 tumors formed rapidly in the control *shGFP*-expressing cells (*N* = 9/10, 90%) (Fig. 4A and Supplementary Fig. 13A). In contrast 2/10 (20%) and 4/8 (50%) tumors formed in the *shPOLE_1* and *shPOLE_2* cohorts, respectively (Fig. 4A and Supplementary Fig. 13A). Of the tumors that formed in *shPOLE_1* and *shPOLE_2* cohorts, we observed re-expression of POLE in tumor explants suggesting that *POLE* knockdown was not sustained in these tumors (Supplementary Fig. 13B). These findings suggest that silencing POLE can suppress tumor formation in vivo. Importantly, of the tumors that grew in the *shPOLE_1* and *shPOLE_2* bearing mice we observed a significantly longer latency period and smaller tumors relative to the *shGFP* control cohort (Fig. 4B), suggesting that POLE suppression can slow the growth of previously formed CIC::DUX4 tumors.

## Discussion

Transcription factor fusion oncoproteins are dominant drivers in human cancers [33]. Through their conserved DNA-binding specificity, TF fusions can regulate multiple target genes simultaneously to control molecular networks and promote malignant potential. Using this insight, we have focused our efforts on identifying the key components that work in concert to promote CIC::DUX4-mediated sarcomagenesis. Specifically, we and others have identified that CIC::DUX4 transcriptionally upregulates *CCNE1*, which compromises the G1/S transition [6] and leads to a dependence on the G2/M checkpoint kinase, WEE1 [7]. WEE1 works as a braking mechanism to slow cellular division that enables effective DNA-repair to occur during S-phase and thus limits mitotic cell death in high-replicative stress states. The current study addresses the question of how CIC::DUX4 sarcoma cells repair DNA prior to mitotic entry and cellular division.

Leveraging our chromatin profiling study, we identified *POLE* as a CIC::DUX4 transcriptional target. Through mechanistic studies we demonstrate that POLE facilitates DNA-repair in CIC::DUX4 sarcomas. Loss of POLE increases DNA damage and leads to p21-mediated cellular senescence. Collectively, these findings suggest that CIC::DUX4 co-opts native cellular machinery to enhance tumor cell growth and survival (Fig. 4C). Specifically, through direct transcriptional upregulation of *CCNE1* and *POLE*, CIC::DUX4 induces a high-replicative stress state that is safeguarded by the G2/M checkpoint kinase, WEE1. Thus, CIC::DUX4 cells escape mitotic catastrophe by delaying mitotic entry (WEE1-dependence) to enable POLE-mediated DNA repair.

Our findings again suggest that CIC::DUX4 can utilize existing native cell-cycle machinery to promote malignant survival. Since CIC::DUX4 can directly upregulate *CCNE1* it makes rational sense to employ the endogenous CCNE/CDK2 inhibitor, p21, to slow cell division and induce senescence [32]. While beyond the scope of this study, we speculate that p21 induces senescence to further delay cellular division once POLE is suppressed. Thus, we now link components of the cell-cycle and DNA replication and repair pathways that are frequently observed to be dysregulated in CIC::DUX4 sarcomas.

Our findings demonstrated that Ewing sarcoma A673 cells, which harbor the EWS::FLI1 fusion, exhibit reduced cell viability upon POLE suppression. Prior studies demonstrated that EWS::FLI1 is associated with high replication stress states, in which CDK12/13 mediates the transcriptional activity of DNA damage repair in fusion-positive Ewing sarcoma. In particular, fusion-positive Ewing sarcoma cells (A673 and TC32) exhibit exquisite sensitivity to THZ531, a CDK12/13 inhibitor, resulting in the downregulation of the homologous recombination (HR) and DNA damage checkpoint genes, including BRCA1, ATR, FANCl, and FANCD2 [34, 35]. Interestingly, *POLE* expression was decreased in A673 and TC32 cells following THZ531 treatment, suggesting that POLE may, in part, contribute to the survival of Ewing sarcoma cells under high replication stress conditions imposed by EWS/FLI-mediated transcription. This should be explored in future studies.

In order to translate our findings with the aim to improve outcomes for CIC::DUX4 patients we need more specific and less toxic therapies. Thus, a limitation of this study is the lack of a POLE specific pharmacologic approach. We used a DNA polymerase inhibitor to demonstrate therapeutic efficacy in our preclinical models, but this is not transferrable to humans. Despite this limitation, we have improved the biological understanding of how CIC::DUX4 survives through high-replicative stress. Altogether we further define the mechanism through which the CIC::DUX4 fusion oncoprotein drives sarcomagenesis. CIC::DUX4 hijacks native cell-cycle checkpoints and DNA repair mechanisms to promote survival. We anticipate that this conserved CCNE/CDK2-POLE-WEE1 axis will serve as a molecular network to therapeutically exploit to improve outcomes for patients with CIC::DUX4 sarcoma.

## Supplementary information

Supplementary Table

Supplementary Figures

## Data Availability

The datasets generated during and/or analysed during the current study are available in the National Center for Biotechnology Information (NCBI) Gene Expression Omnibus database (accession no GSE279467).
