# Supplementary material for: The CIC::DUX4 oncoprotein maintains DNA integrity through direct regulation of the catalytic subunit of DNA polymerase epsilon (POLE)
Source: Oncogene. 2025 Aug 4;44(38):3598–608. doi: 10.1038/s41388-025-03507-9 (PMC12436161; doi:10.1038/s41388-025-03507-9)
Supplement: Supplementary file 1 — Supplementary Table [file 41388_2025_3507_MOESM1_ESM.pdf]

Supplementary Table 1. Differential gene expression comparing NCC_CDS1_X1_C1 cells +/- POLE expression.

ensembl_gene_id	hgnc_symbol	logCPM	PValue	fdr	logFC
ENSG00000130513	GDF15	7.153581393	2.92E-161	4.26E-157	2.03538192
ENSG00000124762	CDKN1A	8.802894747	3.58E-65	2.61E-61	1.0132132
ENSG00000162772	ATF3	5.266281496	1.88E-62	9.17E-59	1.60106533
ENSG00000116717	GADD45A	6.329732822	6.48E-48	2.37E-44	1.18869901
ENSG00000145632	PLK2	4.290976962	1.59E-47	4.66E-44	1.81422131
ENSG00000177606	JUN	6.7229265	1.48E-42	3.60E-39	0.948852
ENSG00000159388	BTG2	6.776369318	8.49E-42	1.77E-38	0.86430889
ENSG00000165891	E2F7	5.921583541	5.39E-41	9.85E-38	1.06592159
ENSG00000106366	SERPINE1	3.393546884	3.53E-30	5.73E-27	1.80896622
ENSG00000181026	AEN	6.630298395	6.43E-29	9.40E-26	0.7825007
ENSG00000174307	PHLDA3	5.792268588	1.95E-28	2.59E-25	0.81821361
ENSG00000184371	CSF1	5.3531612	4.04E-26	4.92E-23	0.89914029
ENSG00000105327	BBC3	6.196825285	7.59E-25	8.54E-22	0.72360849
ENSG00000075426	FOSL2	4.105073522	1.50E-24	1.56E-21	1.20691733
ENSG00000162490	DRAXIN	4.588158532	6.45E-22	6.28E-19	0.94764212
ENSG00000076604	TRAF4	6.649007428	1.94E-21	1.77E-18	0.64958258
ENSG00000126368	NR1D1	4.365628586	2.19E-21	1.88E-18	1.19814422
ENSG00000177084	POLE	10.73816126	6.51E-21	5.29E-18	-0.6080653
ENSG00000265972	TXNIP	5.117560883	1.18E-20	9.09E-18	0.91213301
ENSG00000132510	KDM6B	7.165854488	1.70E-20	1.24E-17	0.59234182
ENSG00000170836	PPM1D	5.895820142	7.43E-20	5.17E-17	0.69298298
ENSG00000135679	MDM2	8.413360197	8.03E-20	5.33E-17	0.53851635
ENSG00000087074	PPP1R15A	5.893869261	1.61E-19	1.02E-16	0.65411968
ENSG00000130766	SESN2	6.055719507	3.25E-18	1.98E-15	0.59915864
ENSG00000239264	TXNDC5	2.577468432	3.83E-18	2.24E-15	2.18421446
ENSG00000164070	HSPA4L	5.702179964	8.63E-18	4.85E-15	0.69428035
ENSG00000009413	REV3L	6.225398647	1.03E-16	5.59E-14	0.66109893
ENSG00000075618	FSCN1	9.741599882	8.94E-16	4.67E-13	-0.551317
ENSG00000026103	FAS	3.095244395	2.25E-15	1.10E-12	1.42655524
ENSG00000153815	CMIP	7.620196776	2.30E-15	1.10E-12	-0.4877571
ENSG00000099194	SCD	8.468177736	2.34E-15	1.10E-12	-0.4971109
ENSG00000144655	CSRNP1	4.973338325	2.83E-15	1.29E-12	0.82576071
ENSG00000101255	TRIB3	6.250517549	5.68E-15	2.51E-12	0.52453465
ENSG00000166851	PLK1	7.238957145	1.05E-14	4.51E-12	-0.4746503
ENSG00000131080	EDA2R	5.527120433	2.29E-14	9.55E-12	0.62716274

ENSG00000149212	SESN3	8.098004575	3.02E-14	1.22E-11	0.46759547
ENSG00000148175	STOM	6.026079416	4.84E-14	1.91E-11	0.52830111
ENSG00000260032	NORAD	8.395817475	6.33E-14	2.43E-11	-0.4366318
ENSG00000106003	LFNG	5.401862796	1.05E-13	3.94E-11	-0.6067302
ENSG00000177595	PIDD1	6.908908742	1.09E-13	4.00E-11	0.59944334
ENSG00000167513	CDT1	7.471897414	3.29E-13	1.17E-10	0.43292117
ENSG00000171848	RRM2	7.46027827	3.98E-13	1.35E-10	0.42644274
ENSG00000234127	TRIM26	6.136590371	3.98E-13	1.35E-10	0.50942676
ENSG00000221869	CEBPD	5.001396285	4.21E-13	1.40E-10	0.62949833
ENSG00000197406	DIO3	10.0817032	5.50E-13	1.74E-10	-0.4641504
ENSG00000168003	SLC3A2	7.932945013	5.63E-13	1.74E-10	0.43115677
ENSG00000186480	INSIG1	6.407809278	5.64E-13	1.74E-10	-0.4752055
ENSG00000130164	LDLR	6.704935346	5.71E-13	1.74E-10	-0.4620317
ENSG00000100647	SUSD6	4.11873738	6.18E-13	1.84E-10	0.84222294
ENSG00000124067	SLC12A4	6.922951521	1.26E-12	3.68E-10	0.48210462
ENSG00000175197	DDIT3	2.744047015	2.92E-12	8.31E-10	1.36704538
ENSG00000054654	SYNE2	6.081168763	2.95E-12	8.31E-10	0.78049019
ENSG00000161513	FDXR	6.51938309	4.34E-12	1.20E-09	0.49343644
ENSG00000071564	TCF3	8.400331079	4.58E-12	1.24E-09	-0.4289817
ENSG00000170734	POLH	6.775519171	6.56E-12	1.74E-09	0.43504635
ENSG00000265354	TIMM23	6.067184639	7.95E-12	2.07E-09	-0.485452
ENSG00000198435	NRARP	7.64854163	1.66E-11	4.25E-09	0.40883239
ENSG00000187837	H1-2	4.851848241	1.71E-11	4.30E-09	-0.6878724
ENSG00000164543	STK17A	4.109930352	1.88E-11	4.65E-09	0.78382944
ENSG00000197903	H2BC12	4.955971505	2.37E-11	5.78E-09	-0.6222772
ENSG00000244509	APOBEC3C	5.731839203	2.43E-11	5.82E-09	0.56760898
ENSG00000225968	ELFN1	6.152876861	3.14E-11	7.41E-09	-0.5071561
ENSG00000168394	TAP1	3.77018099	3.41E-11	7.92E-09	0.84677188
ENSG00000179833	SERTAD2	4.846678222	5.52E-11	1.25E-08	0.59976029
ENSG00000173575	CHD2	7.121413772	5.57E-11	1.25E-08	0.47569114
ENSG00000170385	SLC30A1	6.01981187	7.13E-11	1.58E-08	0.45067342
ENSG00000111846	GCNT2	7.500205063	8.27E-11	1.80E-08	-0.4297617
ENSG00000116678	LEPR	6.825869597	1.02E-10	2.19E-08	0.41126275
ENSG00000059728	MXD1	4.509647865	1.25E-10	2.64E-08	0.64095171
ENSG00000128342	LIF	5.587929183	1.83E-10	3.82E-08	0.51668139
ENSG00000129003	VPS13C	6.322052431	2.06E-10	4.24E-08	0.48325195
ENSG00000078804	TP53INP2	3.650241901	2.44E-10	4.96E-08	0.88706521
ENSG00000272933	TRIM8-DT	3.835044293	3.57E-10	7.15E-08	0.8734954
ENSG00000010165	METTL13	6.039076121	4.68E-10	9.24E-08	-0.4762959
ENSG00000142627	EPHA2	7.348409504	5.23E-10	1.02E-07	0.37976113
ENSG00000156136	DCK	5.579266516	5.66E-10	1.09E-07	-0.4746994

ENSG00000120738	EGR1	2.03671969	6.01E-10	1.13E-07	1.43953397
ENSG00000029993	HMGB3	7.152856328	6.05E-10	1.13E-07	-0.4256721
ENSG00000158055	GRHL3	1.524483659	6.24E-10	1.15E-07	1.77522385
ENSG00000117399	CDC20	7.640411064	6.81E-10	1.25E-07	-0.3950168
ENSG00000103495	MAZ	4.948731372	7.55E-10	1.36E-07	0.62349855
ENSG00000112249	ASCC3	7.429723823	7.70E-10	1.36E-07	0.38964631
ENSG00000171388	APLN	4.218966354	7.75E-10	1.36E-07	0.77644804
ENSG00000134057	CCNB1	7.149954266	1.09E-09	1.89E-07	-0.3817733
ENSG00000047346	ATOSA	5.292047856	1.20E-09	2.06E-07	0.51143816
ENSG00000196586	MYO6	5.286395802	1.33E-09	2.26E-07	0.49731943
ENSG00000138767	CNOT6L	5.962327969	1.43E-09	2.40E-07	0.43226662
ENSG00000113369	ARRDC3	5.246089572	1.46E-09	2.42E-07	0.59532893
ENSG00000125657	TNFSF9	5.002422158	1.49E-09	2.45E-07	0.52989673
ENSG00000184545	DUSP8	4.039917328	1.65E-09	2.68E-07	0.82595896
ENSG00000104856	RELB	4.455547612	2.16E-09	3.47E-07	0.61010659
ENSG00000186897	C1QL4	7.072358002	2.41E-09	3.83E-07	-0.3679358
ENSG00000144224	UBXN4	6.901766276	2.51E-09	3.95E-07	-0.367655
ENSG00000183496	MEX3B	4.439623125	2.64E-09	4.11E-07	0.66568147
ENSG00000251493	FOXD1	5.313589418	2.77E-09	4.27E-07	0.55631003
ENSG00000168079	SCARA5	7.694222986	2.82E-09	4.30E-07	-0.3447083
ENSG00000188229	TUBB4B	10.99237703	3.48E-09	5.24E-07	-0.3894095
ENSG00000135913	USP37	5.264776169	3.54E-09	5.28E-07	0.48257405
ENSG00000171617	ENC1	4.427010915	3.87E-09	5.71E-07	0.66253589
ENSG00000166004	CEP295	4.814102274	3.94E-09	5.76E-07	0.60443771
ENSG00000157741	UBN2	5.151104744	4.59E-09	6.64E-07	0.49704105
ENSG00000102879	CORO1A	7.237104135	5.37E-09	7.70E-07	-0.3503783
ENSG00000171223	JUNB	3.598493918	5.56E-09	7.90E-07	0.78698021
ENSG00000171724	VAT1L	8.855278289	6.29E-09	8.84E-07	-0.3528779
ENSG00000174684	B4GAT1	5.395384521	6.47E-09	9.01E-07	-0.4846327
ENSG00000173846	PLK3	5.812711465	7.45E-09	1.03E-06	0.4622519
ENSG00000197852	INKA2	2.492600012	7.66E-09	1.04E-06	1.22001745
ENSG00000129116	PALLD	7.055613011	7.71E-09	1.04E-06	0.35555516
ENSG00000151247	EIF4E	6.732272278	8.48E-09	1.14E-06	-0.3731261
ENSG00000154898	CCDC144CP	4.568017329	1.13E-08	1.50E-06	0.66646099
ENSG00000100219	XBP1	6.372898418	1.22E-08	1.61E-06	0.37461171
ENSG00000276023	DUSP14	4.939745095	1.28E-08	1.67E-06	0.55420268
ENSG00000055163	CYFIP2	6.760174348	1.41E-08	1.81E-06	0.36418081
ENSG00000129173	E2F8	5.178764059	1.41E-08	1.81E-06	0.48542201
ENSG00000170271	FAXDC2	2.684177832	1.53E-08	1.95E-06	1.0130665
ENSG00000137449	CPEB2	3.301635276	1.60E-08	2.01E-06	0.90285348
ENSG00000104228	TRIM35	6.181868931	1.63E-08	2.04E-06	0.38046542

ENSG00000144426	NBEAL1	4.418302969	1.67E-08	2.07E-06	0.64657991
ENSG00000049130	KITLG	2.148002212	1.72E-08	2.12E-06	1.24760157
ENSG00000132475	H3-3B	9.439527712	1.91E-08	2.33E-06	-0.3519489
ENSG00000139880	CDH24	4.666025403	2.17E-08	2.62E-06	0.53545695
ENSG00000109670	FBXW7	5.481255074	2.42E-08	2.90E-06	0.42863696
ENSG00000074800	ENO1	9.978179112	2.50E-08	2.97E-06	-0.3444436
ENSG00000172432	GTPBP2	6.556497095	3.11E-08	3.67E-06	0.37355018
ENSG00000167553	TUBA1C	7.49467685	3.67E-08	4.29E-06	-0.3989627
ENSG00000172137	CALB2	8.532692286	4.05E-08	4.69E-06	-0.331833
ENSG00000168264	IRF2BP2	7.017080198	4.08E-08	4.69E-06	0.35881976
ENSG00000134333	LDHA	9.73918686	4.14E-08	4.72E-06	-0.3320191
ENSG00000114019	AMOTL2	4.507480268	4.26E-08	4.83E-06	0.67325874
ENSG00000163565	IFI16	7.179082437	4.98E-08	5.60E-06	0.34412715
ENSG00000013016	EHD3	7.203677163	5.03E-08	5.60E-06	-0.339009
ENSG00000167601	AXL	1.515327025	5.06E-08	5.60E-06	1.50236564
ENSG00000135862	LAMC1	7.360739261	5.15E-08	5.66E-06	0.36842299
ENSG00000142871	CCN1	3.648897367	5.38E-08	5.83E-06	0.7144986
ENSG00000255690	TRIL	4.576757472	5.39E-08	5.83E-06	-0.5416914
ENSG00000185432	TMT1A	2.981981391	5.66E-08	6.08E-06	0.95337561
ENSG00000197324	LRP10	6.899788776	5.73E-08	6.11E-06	0.33328199
ENSG00000167552	TUBA1A	8.160281402	5.84E-08	6.16E-06	-0.3253347
ENSG00000175550	DRAP1	7.258616416	5.86E-08	6.16E-06	-0.3655799
ENSG00000181649	PHLDA2	7.651598088	6.16E-08	6.43E-06	-0.355561
ENSG00000143344	RGL1	5.32037234	6.33E-08	6.57E-06	0.43645626
ENSG00000249456	NA	1.689187038	6.91E-08	7.11E-06	1.42779499
ENSG00000076356	PLXNA2	4.036523753	7.33E-08	7.50E-06	0.64071302
ENSG00000165124	SVEP1	4.057496302	7.47E-08	7.54E-06	0.61918272
ENSG00000171988	JMJD1C	6.306595585	7.48E-08	7.54E-06	0.39236915
ENSG00000178401	DNAJC22	5.110807239	7.73E-08	7.74E-06	-0.5209824
ENSG00000284024	MSANTD7	5.651404461	8.00E-08	7.96E-06	0.40945175
ENSG00000259330	INAFM2	5.002787089	1.01E-07	9.94E-06	0.4582119
ENSG00000198589	LRBA	5.755847838	1.02E-07	1.00E-05	0.39723847
ENSG00000111725	PRKAB1	5.085404622	1.06E-07	1.04E-05	0.44867813
ENSG00000102144	PGK1	9.042176464	1.09E-07	1.05E-05	-0.3135112
ENSG00000112984	KIF20A	6.909502437	1.15E-07	1.11E-05	-0.3404457
ENSG00000096070	BRPF3	6.477128203	1.19E-07	1.13E-05	0.3542753
ENSG00000111341	MGP	6.863306921	1.20E-07	1.13E-05	0.34020303
ENSG00000126945	HNRNPH2	6.148756424	1.24E-07	1.17E-05	-0.3903889
ENSG00000015475	BID	7.264512974	1.26E-07	1.18E-05	-0.3178925
ENSG00000100867	DHRS2	0.943614854	1.36E-07	1.27E-05	2.07589793
ENSG00000204899	MZT1	5.984775654	1.47E-07	1.36E-05	-0.3644204

ENSG00000122786	CALD1	7.375742762	1.62E-07	1.49E-05	0.31128016
ENSG00000143476	DTL	6.73852476	1.82E-07	1.67E-05	0.33485025
ENSG00000240891	PLCXD2	2.141224425	1.84E-07	1.67E-05	1.17453014
ENSG00000128923	MINDY2	5.984391986	1.90E-07	1.71E-05	0.40213223
ENSG00000170540	ARL6IP1	7.801776932	1.98E-07	1.77E-05	-0.3090523
ENSG00000181234	TMEM132C	7.260497169	2.05E-07	1.83E-05	-0.3191282
ENSG00000176842	IRX5	3.719471932	2.17E-07	1.92E-05	0.67697869
ENSG00000166801	FAM111A	5.048011512	2.18E-07	1.92E-05	0.56329943
ENSG00000143401	ANP32E	7.501241577	2.31E-07	2.02E-05	-0.3131294
ENSG00000064115	TM7SF3	5.62408085	2.34E-07	2.04E-05	0.39075321
ENSG00000197019	SERTAD1	4.950124769	2.47E-07	2.13E-05	0.46608391
ENSG00000111981	ULBP1	5.40141348	2.48E-07	2.13E-05	0.42681974
ENSG00000116991	SIPA1L2	2.57179261	2.62E-07	2.24E-05	1.07519759
ENSG00000107719	PALD1	6.66092244	2.70E-07	2.30E-05	-0.3274159
ENSG00000188511	MIR3667HG	4.413713667	2.94E-07	2.48E-05	-0.5276808
ENSG00000185361	TNFAIP8L1	5.583176828	3.16E-07	2.65E-05	-0.409222
ENSG00000183853	KIRREL1	6.577749428	3.17E-07	2.65E-05	0.32716976
ENSG00000123983	ACSL3	7.418496635	3.55E-07	2.95E-05	-0.3015663
ENSG00000113742	CPEB4	4.649645255	3.60E-07	2.97E-05	0.5102267
ENSG00000186591	UBE2H	7.220638557	3.91E-07	3.21E-05	0.3072674
ENSG00000134690	CDCA8	6.757793619	3.93E-07	3.21E-05	-0.3188897
ENSG00000146950	SHROOM2	4.937913043	4.19E-07	3.40E-05	-0.4459175
ENSG00000130592	LSP1	5.755330405	4.22E-07	3.40E-05	-0.3702698
ENSG00000117461	PIK3R3	6.653249472	4.31E-07	3.46E-05	0.33564047
ENSG00000151131	NOPCHAP1	5.120982797	4.38E-07	3.50E-05	0.51485342
ENSG00000108561	C1QBP	7.62896392	4.56E-07	3.63E-05	-0.3055997
ENSG00000136826	KLF4	3.110994153	4.86E-07	3.83E-05	0.81888659
ENSG00000136492	BRIP1	5.778249238	4.89E-07	3.83E-05	0.41851412
ENSG00000152527	PLEKHH2	5.331085001	4.91E-07	3.83E-05	0.55041193
ENSG00000141068	KSR1	4.584163344	5.10E-07	3.96E-05	0.51191176
ENSG00000160285	LSS	6.214710561	5.17E-07	4.00E-05	-0.3415528
ENSG00000147526	TACC1	7.018191259	5.46E-07	4.19E-05	0.31672275
ENSG00000144452	ABCA12	2.201717705	5.47E-07	4.19E-05	1.13326312
ENSG00000080546	SESN1	6.545894472	5.72E-07	4.36E-05	0.32928003
ENSG00000188211	NCR3LG1	1.615452107	5.91E-07	4.48E-05	1.28350429
ENSG00000291201	NA	4.582270295	6.26E-07	4.71E-05	0.65262384
ENSG00000104368	PLAT	1.586344993	6.29E-07	4.71E-05	1.3430298
ENSG00000198873	GRK5	6.059530365	6.55E-07	4.89E-05	-0.3456215
ENSG00000114857	NKTR	6.315307775	6.75E-07	5.01E-05	0.46353724
ENSG00000187955	COL14A1	3.761800159	6.82E-07	5.04E-05	0.63955696
ENSG00000111269	CREBL2	6.700049369	6.95E-07	5.11E-05	-0.3505506

ENSG00000051341	POLQ	5.345192801	7.37E-07	5.39E-05	0.40274735
ENSG00000168209	DDIT4	7.218809809	7.46E-07	5.42E-05	0.29839355
ENSG00000128283	CDC42EP1	6.575266331	7.51E-07	5.44E-05	0.31546326
ENSG00000052749	RRP12	4.909584208	7.62E-07	5.49E-05	0.45167388
ENSG00000008300	CELSR3	7.23987706	7.71E-07	5.50E-05	0.30499658
ENSG00000165502	RPL36AL	6.336391797	7.75E-07	5.50E-05	-0.3256729
ENSG00000197558	SSPOP	5.253626744	7.76E-07	5.50E-05	0.49917776
ENSG00000166750	SLFN5	4.756743971	7.97E-07	5.62E-05	0.47411148
ENSG00000112972	HMGCS1	5.435763345	8.00E-07	5.62E-05	-0.3852895
ENSG00000139921	TMX1	6.285936356	8.14E-07	5.69E-05	-0.3398105
ENSG00000099341	PSMD8	8.289310528	8.42E-07	5.86E-05	-0.2996334
ENSG00000164970	FAM219A	6.550452861	8.53E-07	5.91E-05	-0.3199732
ENSG00000185650	ZFP36L1	6.501192712	8.83E-07	6.09E-05	0.31765137
ENSG00000254415	SIGLEC14	-0.755238058	9.27E-07	6.36E-05	3.78760993
ENSG00000051108	HERPUD1	6.817637261	9.42E-07	6.44E-05	-0.3063535
ENSG00000165495	PKNOX2	7.353194454	9.69E-07	6.59E-05	-0.3125175
ENSG00000128944	KNSTRN	5.25134848	9.78E-07	6.62E-05	-0.3939647
ENSG00000067064	IDI1	7.539761848	9.95E-07	6.70E-05	-0.2859009
ENSG00000143799	PARP1	10.15543582	1.01E-06	6.77E-05	-0.3079821
ENSG00000258986	TMEM179	2.471157991	1.03E-06	6.89E-05	0.94100643
ENSG00000060138	YBX3	8.776668559	1.06E-06	7.03E-05	0.28629147
ENSG00000065534	MYLK	7.016320126	1.12E-06	7.36E-05	-0.3008921
ENSG00000179094	PER1	6.504074476	1.12E-06	7.36E-05	0.31313556
ENSG00000159082	SYNJ1	4.738238556	1.12E-06	7.36E-05	0.53914696
ENSG00000168610	STAT3	8.238981077	1.16E-06	7.55E-05	0.28058831
ENSG00000166483	WEE1	6.598087074	1.18E-06	7.68E-05	0.32979704
ENSG00000089685	BIRC5	7.560151961	1.33E-06	8.58E-05	-0.284445
ENSG00000289047	NA	5.159708277	1.38E-06	8.85E-05	0.40826597
ENSG00000055609	KMT2C	6.634279643	1.38E-06	8.85E-05	0.48495021
ENSG00000161800	RACGAP1	6.998399452	1.39E-06	8.85E-05	-0.2962494
ENSG00000198844	ARHGEF15	6.721797874	1.44E-06	9.18E-05	-0.3023228
ENSG00000100097	LGALS1	11.08175585	1.49E-06	9.44E-05	-0.2954033
ENSG00000185813	PCYT2	6.314763725	1.58E-06	9.93E-05	-0.3512179
ENSG00000146592	CREB5	3.539881833	1.68E-06	0.000105	0.80107612
ENSG00000177383	MAGEF1	7.25808624	1.69E-06	0.000105	-0.3018388
ENSG00000197847	SLC22A20P	-0.002930226	1.79E-06	0.00011	2.7057852
ENSG00000142168	SOD1	7.328868928	1.79E-06	0.00011	-0.300777
ENSG00000130054	NALF2	3.031918358	1.80E-06	0.00011	0.80563161
ENSG00000166546	BEAN1	3.673423689	1.80E-06	0.00011	-0.6939158
ENSG00000188191	PRKAR1B	5.906587983	1.81E-06	0.00011	-0.3435238
ENSG00000091140	DLD	6.583606641	1.87E-06	0.000113	-0.3042308

ENSG00000186994	KANK3	5.313231789	1.87E-06	0.000113	0.38055766
ENSG00000109906	ZBTB16	6.076814492	1.89E-06	0.000114	-0.3326679
ENSG00000103260	METRN	7.450427298	1.90E-06	0.000115	-0.2872474
ENSG00000079459	FDFT1	7.326763507	1.94E-06	0.000116	-0.2825246
ENSG00000184584	STING1	4.009671171	1.95E-06	0.000116	0.58111492
ENSG00000054598	FOXC1	7.311328838	2.03E-06	0.000121	0.28453915
ENSG00000174738	NR1D2	4.788233639	2.07E-06	0.000122	0.45309703
ENSG00000174282	ZBTB4	6.245736896	2.08E-06	0.000122	0.33676838
ENSG00000155893	PXYLP1	5.941540351	2.10E-06	0.000123	-0.3348636
ENSG00000204262	COL5A2	5.663669329	2.11E-06	0.000123	0.35378771
ENSG00000116044	NFE2L2	5.662557964	2.11E-06	0.000123	0.42124782
ENSG00000100216	TOMM22	6.692728142	2.18E-06	0.000127	-0.3029912
ENSG00000075461	CACNG4	6.49643476	2.27E-06	0.000131	-0.3144572
ENSG00000136770	DNAJC1	5.089501817	2.38E-06	0.000137	0.40876482
ENSG00000108797	CNTNAP1	8.00523019	2.40E-06	0.000137	0.27773544
ENSG00000187994	RINL	3.943015165	2.41E-06	0.000138	0.55999262
ENSG00000145386	CCNA2	7.279510282	2.42E-06	0.000138	-0.2830502
ENSG00000116584	ARHGEF2	6.049573032	2.45E-06	0.000139	0.33889191
ENSG00000170145	SIK2	5.959759783	2.62E-06	0.000148	0.3492371
ENSG00000291194	NA	5.274576607	2.64E-06	0.000148	0.52970951
ENSG00000120129	DUSP1	5.482091075	2.79E-06	0.000156	0.36601262
ENSG00000134222	PSRC1	5.374913424	2.85E-06	0.000159	-0.3741787
ENSG00000147050	KDM6A	6.293516531	2.86E-06	0.000159	0.31672152
ENSG00000137103	TMEM8B	4.566928273	2.89E-06	0.00016	0.47529522
ENSG00000090776	EFNB1	5.437565691	2.92E-06	0.000161	0.36577666
ENSG00000078900	TP73	4.401726201	2.93E-06	0.000161	0.48386174
ENSG00000184635	ZNF93	4.958319246	2.98E-06	0.000163	0.41559035
ENSG00000261371	PECAM1	7.100208492	3.14E-06	0.000171	-0.2889686
ENSG00000114698	PLSCR4	6.286255499	3.19E-06	0.000173	0.31401429
ENSG00000285756	NA	5.811777816	3.20E-06	0.000173	0.33570699
ENSG00000076003	MCM6	7.726383025	3.21E-06	0.000173	-0.2698049
ENSG00000171314	PGAM1	8.436468526	3.25E-06	0.000175	-0.2837769
ENSG00000077782	FGFR1	9.66019275	3.36E-06	0.00018	-0.272401
ENSG00000153208	MERTK	5.101781104	3.38E-06	0.00018	0.40659158
ENSG00000100522	GNPNAT1	4.754385751	3.39E-06	0.00018	-0.4315154
ENSG00000185418	TARS3	4.787467072	3.43E-06	0.000182	-0.4490639
ENSG00000188747	NOXA1	5.329939412	3.58E-06	0.000189	0.46029452
ENSG00000135114	OASL	2.26062555	3.79E-06	0.000199	0.96300853
ENSG00000154767	XPC	5.500162016	4.09E-06	0.000214	0.36025432
ENSG00000139044	B4GALNT3	6.922599697	4.31E-06	0.000225	-0.3055329
ENSG00000157613	CREB3L1	9.416273247	4.41E-06	0.000229	-0.2711004

ENSG00000123989	CHPF	6.091907656	4.42E-06	0.000229	0.32604572
ENSG00000229807	XIST	7.837647311	4.46E-06	0.000231	0.58164224
ENSG00000116539	ASH1L	6.635696246	4.56E-06	0.000233	0.32939787
ENSG00000115884	SDC1	4.188522234	4.57E-06	0.000233	0.536996
ENSG00000127481	UBR4	6.952781881	4.58E-06	0.000233	0.36054872
ENSG00000168564	CDKN2AIP	4.579622321	4.58E-06	0.000233	0.44616023
ENSG00000022567	SLC45A4	2.631951931	4.60E-06	0.000233	0.86609983
ENSG00000129355	CDKN2D	5.606333347	4.69E-06	0.000237	-0.3508861
ENSG00000135074	ADAM19	6.486012739	4.80E-06	0.000242	-0.2954202
ENSG00000129195	PIMREG	7.809890389	4.90E-06	0.000246	-0.2639312
ENSG00000138413	IDH1	5.888835855	5.05E-06	0.000253	-0.3252835
ENSG00000164331	ANKRA2	4.000082989	5.16E-06	0.000257	0.55672741
ENSG00000196152	ZNF79	3.995073275	5.26E-06	0.000261	0.57289384
ENSG00000119541	VPS4B	6.140799927	5.30E-06	0.000263	-0.3244956
ENSG00000054148	PHPT1	6.538120602	5.40E-06	0.000267	0.3189958
ENSG00000113719	ERGIC1	7.648577222	5.44E-06	0.000267	-0.2755963
ENSG00000245532	NEAT1	6.78020046	5.63E-06	0.000276	0.58688541
ENSG00000184185	KCNJ12	6.565526892	5.66E-06	0.000276	-0.3225998
ENSG00000163931	TKT	8.619520926	5.68E-06	0.000277	-0.3164019
ENSG00000134574	DDB2	6.81494677	5.92E-06	0.000287	0.28814374
ENSG00000130449	ZSWIM6	6.177058013	5.93E-06	0.000287	0.31182427
ENSG00000188566	NDOR1	6.927416078	5.96E-06	0.000287	0.27970179
ENSG00000138758	SEPTIN11	10.02491753	5.99E-06	0.000288	-0.2709556
ENSG00000182718	ANXA2	9.803473444	6.24E-06	0.000299	-0.2810562
ENSG00000189056	RELN	3.902924097	6.27E-06	0.000299	0.54394055
ENSG00000085491	SLC25A24	6.046373881	6.36E-06	0.000303	-0.3101025
ENSG00000111669	TPI1	9.622675034	6.61E-06	0.000314	-0.2724411
ENSG00000141682	PMAIP1	2.187499197	6.69E-06	0.000317	1.00509943
ENSG00000166831	RBPMS2	3.532946025	6.78E-06	0.00032	0.62059701
ENSG00000186298	PPP1CC	8.641033955	6.83E-06	0.000321	-0.2737187
ENSG00000052802	MSMO1	4.478223822	6.91E-06	0.000323	-0.4735195
ENSG00000248429	GASK1B-AS1	1.87010922	6.92E-06	0.000323	1.03003615
ENSG00000116574	RHOU	3.87766226	6.94E-06	0.000323	0.54788787
ENSG00000005189	REXO5	3.357499717	7.11E-06	0.000329	0.64605096
ENSG00000166398	GARRE1	4.691464086	7.11E-06	0.000329	0.44677565
ENSG00000242732	RTL5	3.317353669	7.13E-06	0.000329	0.68879037
ENSG00000186063	AIDA	7.518253446	7.51E-06	0.000345	-0.2645448
ENSG00000111788	DDX12B	3.928343255	7.55E-06	0.000346	0.55542776
ENSG00000213638	ADAT3	3.306697009	7.60E-06	0.000347	-0.6626979
ENSG00000065308	TRAM2	6.069855041	7.65E-06	0.000348	0.3061157
ENSG00000088832	FKBP1A	7.967289949	7.68E-06	0.000349	-0.2700815

ENSG00000123908	AGO2	8.02257195	7.73E-06	0.000349	0.31909792
ENSG00000110871	COQ5	4.932365815	7.74E-06	0.000349	-0.4013506
ENSG00000146083	RNF44	6.04317372	8.23E-06	0.00037	0.33368611
ENSG00000113583	C5orf15	5.92280028	8.38E-06	0.000376	-0.3164473
ENSG00000163635	ATXN7	5.819735415	8.45E-06	0.000377	0.37347714
ENSG00000130702	LAMA5	6.928749744	8.46E-06	0.000377	0.2942964
ENSG00000177943	MAMDC4	7.758960757	8.49E-06	0.000377	0.43926958
ENSG00000226762	LINC02668	5.322656681	8.53E-06	0.000378	-0.3739743
ENSG00000075213	SEMA3A	3.654955134	8.60E-06	0.00038	0.59849692
ENSG00000145362	ANK2	3.895930315	9.18E-06	0.000404	0.55791982
ENSG00000145050	MANF	5.693749835	9.41E-06	0.000413	-0.3265173
ENSG00000143870	PDIA6	6.92258832	9.48E-06	0.000415	-0.2833199
ENSG00000042445	RETSAT	5.148233153	9.70E-06	0.000423	0.37360185
ENSG00000113161	HMGCR	6.577724603	9.76E-06	0.000424	-0.3112405
ENSG00000172216	CEBPB	5.119718424	9.99E-06	0.000433	0.37481614
ENSG00000099817	POLR2E	8.111642736	1.02E-05	0.000441	-0.3082096
ENSG00000069399	BCL3	7.28969932	1.02E-05	0.00044	0.26537557
ENSG00000152104	PTPN14	4.422915464	1.06E-05	0.000456	0.46762302
ENSG00000132622	HSPA12B	8.315755955	1.09E-05	0.000469	-0.256391
ENSG00000272405	NA	1.572423259	1.12E-05	0.00048	1.18220543
ENSG00000102024	PLS3	7.386081179	1.14E-05	0.000487	-0.2635404
ENSG00000104549	SQLE	5.511680865	1.15E-05	0.000488	-0.3335403
ENSG00000071967	CYBRD1	6.603734361	1.17E-05	0.000495	0.27950261
ENSG00000175105	ZNF654	5.328595931	1.18E-05	0.000497	0.35267756
ENSG00000135334	AKIRIN2	5.905993068	1.19E-05	0.000503	-0.313656
ENSG00000162738	VANGL2	4.513519972	1.20E-05	0.000505	0.44311501
ENSG00000118503	TNFAIP3	3.85177602	1.20E-05	0.000503	0.54374938
ENSG00000172889	EGFL7	7.926269286	1.24E-05	0.000518	-0.2763116
ENSG00000103657	HERC1	6.174574816	1.27E-05	0.000529	0.35532842
ENSG00000166986	MARS1	7.27767133	1.30E-05	0.00054	-0.2608551
ENSG00000108100	CCNY	7.046666821	1.30E-05	0.000538	-0.273861
ENSG00000132669	RIN2	6.751977865	1.31E-05	0.000541	-0.2799988
ENSG00000069020	MAST4	0.588440683	1.34E-05	0.000553	1.73823391
ENSG00000176692	FOXC2	7.839254102	1.35E-05	0.000553	0.29841229
ENSG00000188486	H2AX	9.169560896	1.38E-05	0.000565	-0.2664039
ENSG00000274523	RCC1L	6.633961654	1.39E-05	0.000568	-0.3072464
ENSG00000186529	CYP4F3	-0.527383106	1.39E-05	0.000566	2.74583876
ENSG00000152818	UTRN	8.165896387	1.41E-05	0.000572	0.29270451
ENSG00000176171	BNIP3	6.668351408	1.41E-05	0.000572	-0.2811211
ENSG00000131473	ACLY	8.576121627	1.48E-05	0.000599	-0.2724227
ENSG00000116704	SLC35D1	4.802248878	1.52E-05	0.000612	0.43391961

ENSG00000162924	REL	2.601160435	1.53E-05	0.000613	0.79012462
ENSG00000156232	WHAMM	4.89907745	1.58E-05	0.000634	0.38238844
ENSG00000103507	BCKDK	6.645015291	1.60E-05	0.000638	-0.2859391
ENSG00000125743	SNRPD2	7.950556686	1.60E-05	0.000638	-0.2506584
ENSG00000166987	MBD6	6.76140229	1.67E-05	0.000662	0.280606
ENSG00000127603	MACF1	7.256028708	1.67E-05	0.000662	0.38270678
ENSG00000130204	TOMM40	9.382818145	1.70E-05	0.000671	-0.2678133
ENSG00000168621	GDNF	0.262198243	1.72E-05	0.000677	1.96187826
ENSG00000104960	PTOV1	5.730903152	1.73E-05	0.000679	0.57241571
ENSG00000159792	PSKH1	5.660434337	1.77E-05	0.000692	0.31639542
ENSG00000177469	CAVIN1	8.881372733	1.81E-05	0.000706	-0.2638882
ENSG00000008311	AASS	1.218419714	1.81E-05	0.000706	1.24968091
ENSG00000104976	SNAPC2	6.980679521	1.82E-05	0.000707	-0.2743878
ENSG00000172893	DHCR7	5.568350652	1.86E-05	0.00072	-0.4223242
ENSG00000173230	GOLGB1	6.718002736	1.93E-05	0.000746	0.356903
ENSG00000152117	SMPD4BP	4.710833657	1.96E-05	0.000755	0.45329902
ENSG00000145390	USP53	4.131817439	1.96E-05	0.000755	0.48303155
ENSG00000179041	RRS1	6.068374169	1.98E-05	0.000761	-0.2980356
ENSG00000171219	CDC42BPG	0.88743623	2.03E-05	0.000776	1.58054087
ENSG00000156515	HK1	8.700467071	2.03E-05	0.000776	-0.2520118
ENSG00000100439	ABHD4	4.203544997	2.06E-05	0.000785	0.51580444
ENSG00000005810	MYCBP2	7.733295339	2.09E-05	0.000795	0.31964462
ENSG00000132341	RAN	9.240613296	2.10E-05	0.000795	-0.2688835
ENSG00000024526	DEPDC1	5.540881909	2.11E-05	0.000795	-0.3224757
ENSG00000287263	NA	1.524096343	2.11E-05	0.000795	1.11505362
ENSG00000100100	PIK3IP1	3.383605494	2.12E-05	0.000796	0.70019947
ENSG00000180828	BHLHE22	6.598994865	2.13E-05	0.000797	-0.2786058
ENSG00000185686	PRAME	6.971869081	2.15E-05	0.000804	-0.26485
ENSG00000120693	SMAD9	4.443671095	2.18E-05	0.000813	0.44444754
ENSG00000147649	MTDH	8.253327126	2.25E-05	0.000838	-0.2493793
ENSG00000135912	TTLL4	5.29687463	2.27E-05	0.00084	0.34140863
ENSG00000221843	SPATA31H1	2.930117504	2.27E-05	0.00084	0.71470092
ENSG00000169692	AGPAT2	8.118286074	2.28E-05	0.00084	-0.25755
ENSG00000170345	FOS	5.587059666	2.31E-05	0.000849	0.32200894
ENSG00000064687	ABCA7	5.572367419	2.51E-05	0.000923	0.41508651
ENSG00000087085	ACHE	2.037768425	2.58E-05	0.000946	0.97279412
ENSG00000101670	LIPG	7.696371781	2.69E-05	0.000981	-0.2504813
ENSG00000196576	PLXNB2	9.387954328	2.73E-05	0.000994	0.24723124
ENSG00000079385	CEACAM1	0.65563624	2.81E-05	0.001022	1.59181398
ENSG00000183207	RUVBL2	7.500070626	2.83E-05	0.001027	-0.2500513
ENSG00000176531	PHLDB3	3.851588114	2.84E-05	0.001028	0.60917022

ENSG00000139734	DIAPH3	5.647528803	2.97E-05	0.00107	0.31155584
ENSG00000187239	FNBP1	7.722000689	2.98E-05	0.001072	-0.2721171
ENSG00000111229	ARPC3	6.860496802	2.98E-05	0.001072	-0.2596691
ENSG00000167232	ZNF91	4.257265101	3.11E-05	0.001115	0.5034624
ENSG00000178718	RPP25	5.782792188	3.14E-05	0.001121	-0.3008197
ENSG00000211460	TSN	6.917616009	3.18E-05	0.001133	-0.25897
ENSG00000141232	TOB1	7.109337567	3.19E-05	0.001135	0.25363133
ENSG00000132603	NIP7	5.59046106	3.23E-05	0.001147	-0.3570335
ENSG00000177098	SCN4B	4.920234436	3.38E-05	0.001195	0.36672721
ENSG00000165688	PMPCA	7.323629535	3.44E-05	0.001214	-0.2471546
ENSG00000183087	GAS6	6.076757332	3.53E-05	0.001244	0.28859924
ENSG00000100029	PES1	6.764321889	3.58E-05	0.001259	-0.269163
ENSG00000169118	CSNK1G1	5.064846294	3.63E-05	0.001274	0.35664027
ENSG00000153989	NUS1	6.467954951	3.64E-05	0.001274	-0.2965549
ENSG00000177156	TALDO1	7.70668075	3.72E-05	0.001298	-0.2468061
ENSG00000182022	CHST15	6.481255723	3.78E-05	0.001315	-0.2808195
ENSG00000150938	CRIM1	4.830575672	3.93E-05	0.001361	0.39523197
ENSG00000078674	PCM1	7.377929931	3.93E-05	0.001361	0.26431962
ENSG00000099204	ABLIM1	4.599031146	3.95E-05	0.001365	0.4012705
ENSG00000131711	MAP1B	5.948618274	3.99E-05	0.001375	0.42231909
ENSG00000101624	CEP76	3.231423948	4.00E-05	0.001376	0.61041874
ENSG00000117525	F3	2.471436505	4.13E-05	0.001416	0.7718927
ENSG00000134824	FADS2	4.798319821	4.15E-05	0.001418	-0.3812711
ENSG00000105254	TBCB	7.629726263	4.15E-05	0.001418	-0.2519861
ENSG00000234745	HLA-B	2.982815799	4.16E-05	0.001419	0.69877415
ENSG00000184254	ALDH1A3	7.061422568	4.19E-05	0.001425	-0.2599073
ENSG00000126457	PRMT1	8.192448754	4.25E-05	0.001441	-0.2612303
ENSG00000126778	SIX1	6.148416143	4.26E-05	0.001442	0.30808645
ENSG00000182541	LIMK2	5.597094339	4.31E-05	0.001455	0.33193277
ENSG00000105173	CCNE1	7.663935803	4.33E-05	0.001459	0.2387353
ENSG00000271447	MMP28	4.24757064	4.38E-05	0.001472	0.44328606
ENSG00000181444	ZNF467	4.89295398	4.50E-05	0.00151	0.3700776
ENSG00000152229	PSTPIP2	4.656094504	4.57E-05	0.001528	0.39575657
ENSG00000150753	CCT5	8.007909729	4.58E-05	0.00153	-0.252367
ENSG00000118762	PKD2	6.70115496	4.60E-05	0.001531	0.28084892
ENSG00000169100	SLC25A6	10.1314693	4.66E-05	0.001548	-0.2646894
ENSG00000167202	TBC1D2B	5.573951708	4.68E-05	0.001551	0.33269978
ENSG00000095564	BTAF1	6.625761502	4.74E-05	0.001568	0.30546263
ENSG00000140479	PCSK6	6.347220557	4.76E-05	0.001569	-0.2683882
ENSG00000147536	GINS4	5.43899988	4.82E-05	0.001588	0.31964511
ENSG00000120889	TNFRSF10B	8.881996734	4.85E-05	0.001593	0.23502624

ENSG00000135404	CD63	7.70746972	4.95E-05	0.001621	-0.2443922
ENSG00000139292	LGR5	4.263659477	5.00E-05	0.001636	0.45599311
ENSG00000179406	LINC00174	2.389446679	5.06E-05	0.001651	0.90530363
ENSG00000169710	FASN	10.03638047	5.07E-05	0.001651	-0.2529674
ENSG00000183248	PRR36	5.028051825	5.16E-05	0.001675	0.36359583
ENSG00000154096	THY1	8.592625854	5.17E-05	0.001676	-0.2392796
ENSG00000161642	ZNF385A	4.971330706	5.19E-05	0.001679	0.3544299
ENSG00000141522	ARHGDIA	8.791630562	5.25E-05	0.001695	-0.2596672
ENSG00000145912	NHP2	5.666130533	5.29E-05	0.001704	-0.3033714
ENSG00000158985	CDC42SE2	5.674108318	5.35E-05	0.00172	-0.3213856
ENSG00000116133	DHCR24	7.177605228	5.43E-05	0.00174	-0.2675509
ENSG00000135723	FHOD1	3.198872228	5.44E-05	0.00174	0.65731938
ENSG00000179331	RAB39A	2.139528828	5.52E-05	0.00176	0.83643037
ENSG00000047634	SCML1	4.666526261	5.57E-05	0.001774	0.44280429
ENSG00000244586	WNT5A-AS1	4.823961872	5.60E-05	0.00178	-0.3644765
ENSG00000134375	TIMM17A	5.965679829	5.66E-05	0.001794	-0.2815354
ENSG00000118058	KMT2A	6.742102347	5.70E-05	0.001804	0.38013412
ENSG00000136010	ALDH1L2	4.485060852	5.72E-05	0.001807	0.40198074
ENSG00000198736	MSRB1	5.087208542	5.76E-05	0.001815	-0.3542218
ENSG00000140199	SLC12A6	4.693427567	5.85E-05	0.00184	0.43311486
ENSG00000196730	DAPK1	4.412629402	5.89E-05	0.001845	0.41930095
ENSG00000047457	CP	2.432864189	5.90E-05	0.001845	0.80579797
ENSG00000171530	TBCA	6.392625609	6.02E-05	0.001874	-0.2712014
ENSG00000063127	SLC6A16	2.239059312	6.02E-05	0.001874	0.82769536
ENSG00000197647	ZNF433	0.379139171	6.03E-05	0.001874	1.61127161
ENSG00000154822	PLCL2	1.81829326	6.06E-05	0.001881	0.94963498
ENSG00000169635	HIC2	5.532843271	6.07E-05	0.001881	0.31122007
ENSG00000275342	PRAG1	6.233736625	6.13E-05	0.001895	0.28624814
ENSG00000169679	BUB1	6.556825936	6.23E-05	0.00192	-0.257357
ENSG00000149257	SERPINH1	9.422681277	6.24E-05	0.001921	-0.2656589
ENSG00000176101	SSNA1	8.009713312	6.35E-05	0.00195	-0.2558597
ENSG00000198833	UBE2J1	7.787791992	6.43E-05	0.001972	-0.2305013
ENSG00000101104	PABPC1L	2.739356184	6.46E-05	0.001976	0.84720807
ENSG00000004399	PLXND1	10.76826594	6.49E-05	0.001982	-0.2414738
ENSG00000065357	DGKA	4.427024237	6.63E-05	0.002019	0.41127707
ENSG00000277791	PSMB3	6.565306048	6.68E-05	0.00203	-0.2656441
ENSG00000056487	PHF21B	2.797491711	6.74E-05	0.002043	0.68112193
ENSG00000108219	TSPAN14	8.274951327	6.83E-05	0.002068	-0.2347558
ENSG00000272888	CHASERR	3.944483245	6.87E-05	0.002075	0.61839305
ENSG00000110888	CAPRIN2	2.744422082	6.99E-05	0.002107	0.76732032
ENSG00000174371	EXO1	5.688266311	7.01E-05	0.002108	0.32841795

ENSG00000100519	PSMC6	6.12981998	7.04E-05	0.002112	-0.2815382
ENSG00000149654	CDH22	4.177380609	7.10E-05	0.002126	-0.4586063
ENSG00000083857	FAT1	9.30894215	7.37E-05	0.002203	0.32628606
ENSG00000146535	GNA12	8.020290512	7.43E-05	0.002218	-0.2295554
ENSG00000116260	QSOX1	7.434773518	7.46E-05	0.002218	-0.251039
ENSG00000157764	BRAF	5.106570159	7.47E-05	0.002218	0.35062413
ENSG00000137267	TUBB2A	5.98501513	7.61E-05	0.002255	-0.2895294
ENSG00000196295	GARS1-DT	3.649225473	7.66E-05	0.002265	0.62421887
ENSG00000162599	NFIA	6.066001286	7.71E-05	0.002276	0.27938578
ENSG00000114861	FOXP1	4.629314531	7.73E-05	0.002276	0.40507155
ENSG00000133131	MORC4	4.874934195	7.74E-05	0.002276	0.3871283
ENSG00000185651	UBE2L3	7.577666025	7.84E-05	0.002301	-0.2493525
ENSG00000123384	LRP1	8.782502898	7.87E-05	0.002305	0.29028425
ENSG00000177200	CHD9	6.541390742	7.94E-05	0.00232	0.28977086
ENSG00000180998	GPR137C	3.700005899	7.97E-05	0.002321	0.61341547
ENSG00000277196	NA	1.236315042	7.97E-05	0.002321	1.14197054
ENSG00000125944	HNRNPR	7.623775579	8.11E-05	0.002357	-0.230259
ENSG00000005513	SOX8	5.09320774	8.33E-05	0.002416	-0.3373725
ENSG00000167548	KMT2D	8.18790616	8.47E-05	0.002453	0.34768559
ENSG00000144959	NCEH1	3.36396996	8.51E-05	0.002458	0.57198193
ENSG00000185668	POU3F1	1.53867447	8.58E-05	0.002466	1.1623998
ENSG00000196419	XRCC6	9.108856563	8.58E-05	0.002466	-0.2446399
ENSG00000072736	NFATC3	5.327481393	8.60E-05	0.002466	0.32755073
ENSG00000083720	OXCT1	6.102566966	8.63E-05	0.002466	-0.2764988
ENSG00000010818	HIVEP2	2.209801747	8.63E-05	0.002466	0.80546864
ENSG00000149792	MRPL49	6.438688126	8.66E-05	0.002466	0.25650918
ENSG00000060656	PTPRU	5.394011456	8.67E-05	0.002466	0.30823412
ENSG00000162783	IER5	6.933046054	8.69E-05	0.002466	0.26367793
ENSG00000139645	ANKRD52	7.603398245	8.69E-05	0.002466	0.24979688
ENSG00000109062	NHERF1	5.749034318	8.71E-05	0.002468	-0.2899288
ENSG00000182752	PAPPA	4.031728637	8.77E-05	0.002479	0.52292971
ENSG00000046604	DSG2	1.279907726	8.87E-05	0.002503	1.12230351
ENSG00000100401	RANGAP1	7.478602408	8.89E-05	0.002504	-0.2332516
ENSG00000173064	HECTD4	6.638472305	8.99E-05	0.002526	0.30680617
ENSG00000167508	MVD	5.880954853	9.05E-05	0.002539	-0.2795173
ENSG00000189120	SP6	2.070109804	9.09E-05	0.002544	0.89514155
ENSG00000140350	ANP32A	7.860086731	9.15E-05	0.002556	-0.2359418
ENSG00000136367	ZFHX2	2.96362396	9.17E-05	0.002557	0.69153552
ENSG00000115461	IGFBP5	7.68594749	9.23E-05	0.002569	-0.2497982
ENSG00000089220	PEBP1	7.981264745	9.26E-05	0.002574	-0.2497021
ENSG00000103647	CORO2B	5.665323006	9.29E-05	0.002576	-0.2935331

ENSG00000121068	TBX2	3.094290791	9.33E-05	0.002584	0.67483961
ENSG00000006327	TNFRSF12A	6.268911631	9.43E-05	0.002605	0.26729533
ENSG00000105613	MAST1	5.25335938	9.51E-05	0.002623	0.33576795
ENSG00000090238	YPEL3	4.103449315	9.53E-05	0.002624	0.46124952
ENSG00000135116	HRK	-1.112675729	9.74E-05	0.002675	3.29679542
ENSG00000108106	UBE2S	8.439499674	9.79E-05	0.002685	-0.2352491
ENSG00000117450	PRDX1	7.355804626	9.86E-05	0.0027	-0.2578124
ENSG00000034510	TMSB10	8.799772285	9.98E-05	0.002727	-0.2321081
ENSG00000108518	PFN1	9.729402799	0.0001006	0.00274	-0.2338635
ENSG00000121966	CXCR4	1.706977413	0.0001006	0.00274	0.94653135
ENSG00000197894	ADH5	7.073404597	0.0001014	0.002755	-0.2414902
ENSG00000185825	BCAP31	7.241068375	0.0001018	0.00276	-0.2426815
ENSG00000112242	E2F3	6.118771501	0.0001022	0.002767	0.26429806
ENSG00000123131	PRDX4	5.324949033	0.0001024	0.002767	-0.3263003
ENSG00000049245	VAMP3	5.938523208	0.0001031	0.00278	-0.2728513
ENSG00000070367	EXOC5	6.815890385	0.0001062	0.002858	-0.2405949
ENSG00000172531	PPP1CA	8.07894566	0.0001069	0.002873	-0.2329689
ENSG00000106086	PLEKHA8	0.863541932	0.0001081	0.002899	1.33666033
ENSG00000065978	YBX1	10.36059113	0.0001091	0.002921	-0.2510929
ENSG00000133424	LARGE1	6.765972844	0.0001099	0.002938	-0.2715868
ENSG00000132432	SEC61G	5.958008868	0.0001105	0.002941	-0.2704199
ENSG00000163251	FZD5	3.869116802	0.0001108	0.002941	0.491449
ENSG00000130522	JUND	7.649490121	0.0001109	0.002941	0.23912468
ENSG00000143155	TIPRL	5.563280201	0.000111	0.002941	-0.2923882
ENSG00000105750	ZNF85	3.929215065	0.0001111	0.002941	0.47982054
ENSG00000133393	CEP20	5.711124552	0.0001117	0.002946	-0.2986125
ENSG00000153179	RASSF3	6.118396525	0.0001118	0.002946	-0.2943102
ENSG00000161509	GRIN2C	3.647933192	0.0001119	0.002946	0.78504196
ENSG00000135144	DTX1	4.61775482	0.0001132	0.002972	-0.3733457
ENSG00000263465	SRSF8	5.144769167	0.0001133	0.002972	0.31938317
ENSG00000157106	SMG1	7.438600483	0.0001136	0.002977	0.32753408
ENSG00000103855	CD276	9.030909293	0.0001147	0.003	-0.2276445
ENSG00000130741	EIF2S3	8.339360794	0.0001159	0.003025	-0.2360115
ENSG00000112559	MDFI	7.706047578	0.0001183	0.003082	-0.2278688
ENSG00000155368	DBI	5.79255885	0.000119	0.003096	-0.2754079
ENSG00000011485	PPP5C	8.617153933	0.0001213	0.00315	-0.2259789
ENSG00000260912	NA	3.760535261	0.000124	0.003215	0.49631951
ENSG00000168010	ATG16L2	3.111737926	0.0001272	0.003289	0.67286884
ENSG00000130382	MLLT1	7.36526872	0.0001274	0.003289	-0.2374376
ENSG00000005801	ZNF195	5.635334217	0.0001314	0.003378	0.35789892
ENSG00000273018	FAM106A	3.32443887	0.0001314	0.003378	0.55704779

ENSG00000170606	HSPA4	7.713093427	0.0001315	0.003378	-0.2324726
ENSG00000118689	FOXO3	6.3674391	0.0001317	0.003378	0.2650005
ENSG00000156802	ATAD2	7.834632	0.0001342	0.003436	0.2223823
ENSG00000126787	DLGAP5	6.176601526	0.0001366	0.003491	-0.2658065
ENSG00000119185	ITGB1BP1	6.090955982	0.0001382	0.003525	-0.2647722
ENSG00000136153	LMO7	2.273914512	0.0001393	0.003545	0.79140799
ENSG00000235513	L3MBTL2-AS1	0.877837693	0.0001395	0.003545	1.51264715
ENSG00000177599	ZNF491	2.166960954	0.00014	0.003549	0.79493759
ENSG00000130787	HIP1R	4.180392302	0.0001401	0.003549	0.43101112
ENSG00000072310	SREBF1	8.376143507	0.000141	0.003565	-0.2180136
ENSG00000105639	JAK3	3.907299594	0.0001425	0.003598	0.46776332
ENSG00000128965	CHAC1	3.542150375	0.0001428	0.003599	0.54049408
ENSG00000104381	GDAP1	3.376549189	0.0001431	0.003599	0.5603702
ENSG00000105483	CARD8	3.722478759	0.0001433	0.003599	0.522336
ENSG00000277443	MARCKS	8.74505301	0.0001448	0.003631	0.23569509
ENSG00000160179	ABCG1	5.380069707	0.0001463	0.003663	-0.3057401
ENSG00000144596	GRIP2	0.313913656	0.0001488	0.003714	1.62151573
ENSG00000104765	BNIP3L	7.27010009	0.0001489	0.003714	-0.2258181
ENSG00000170776	AKAP13	5.35831724	0.00015	0.003736	0.32813086
ENSG00000179588	ZFPM1	5.233189743	0.0001506	0.003741	0.32378692
ENSG00000175063	UBE2C	7.136608663	0.0001508	0.003741	-0.2372516
ENSG00000099800	TIMM13	6.532244603	0.0001512	0.003745	-0.2825852
ENSG00000172890	NADSYN1	5.732481753	0.0001514	0.003745	0.32649709
ENSG00000100344	PNPLA3	4.928440766	0.0001544	0.003813	-0.3320605
ENSG00000173674	EIF1AX	7.591150236	0.0001552	0.003827	-0.2378416
ENSG00000211584	SLC48A1	3.447689167	0.0001564	0.00385	0.53064423
ENSG00000185736	ADARB2	4.266868356	0.0001567	0.003851	-0.4094944
ENSG00000102531	FNDC3A	5.992443166	0.0001582	0.003879	0.26170674
ENSG00000100814	CCNB1IP1	4.977012087	0.0001611	0.003946	0.33447554
ENSG00000136717	BIN1	5.237901141	0.0001652	0.004032	-0.3354751
ENSG00000108479	GALK1	6.865097828	0.0001652	0.004032	-0.2379947
ENSG00000104517	UBR5	7.951388434	0.0001663	0.004052	0.2351723
ENSG00000094804	CDC6	7.06519451	0.0001691	0.004112	0.22846359
ENSG00000171723	GPHN	5.967831422	0.0001697	0.00412	0.26188024
ENSG00000108854	SMURF2	5.144247599	0.0001705	0.004132	0.32478425
ENSG00000185818	NAT8L	5.939141384	0.0001715	0.00415	-0.3030475
ENSG00000141867	BRD4	7.921513888	0.0001725	0.004168	0.24934412
ENSG00000099860	GADD45B	2.903788588	0.0001734	0.004179	0.60773958
ENSG00000172244	C5orf34	2.964081322	0.0001735	0.004179	0.60446772
ENSG00000139835	GRTP1	5.486059165	0.000175	0.004206	-0.2996052
ENSG00000155876	RRAGA	6.790472823	0.0001781	0.004274	-0.2388697

ENSG00000177076	ACER2	4.682307647	0.0001791	0.004289	0.37811114
ENSG00000186638	KIF24	4.23613608	0.0001793	0.004289	0.40600964
ENSG00000106236	NPTX2	4.738443261	0.0001815	0.004335	-0.3500161
ENSG00000095397	WHRN	0.560767953	0.000184	0.004388	1.54292965
ENSG00000146247	PHIP	6.701917714	0.0001865	0.00444	0.3476296
ENSG00000249859	PVT1	3.709202427	0.0001874	0.004454	0.5166574
ENSG00000096384	HSP90AB1	10.84643286	0.0001891	0.004488	-0.2350289
ENSG00000101182	PSMA7	7.916124493	0.0001902	0.004505	-0.2285653
ENSG00000168092	PAFAH1B2	7.437664469	0.0001907	0.004511	-0.2315147
ENSG00000172809	RPL38	7.495978905	0.0001922	0.004533	-0.2233601
ENSG00000162063	CCNF	7.01015392	0.0001923	0.004533	-0.2271128
ENSG00000197747	S100A10	9.857146912	0.0001938	0.004561	-0.224252
ENSG00000179361	ARID3B	4.789503498	0.0001945	0.004571	0.35511194
ENSG00000105325	FZR1	6.902332461	0.0001954	0.004585	-0.232977
ENSG00000120709	FAM53C	6.616791711	0.0001964	0.004602	0.24075977
ENSG00000086065	CHMP5	5.818229169	0.0001984	0.00464	-0.2857181
ENSG00000171161	ZNF672	6.37600531	0.000205	0.004784	0.25042299
ENSG00000106211	HSPB1	9.891334524	0.0002054	0.004784	-0.2321829
ENSG00000276903	H2AC16	-0.135109507	0.0002056	0.004784	-1.8317855
ENSG00000083290	ULK2	5.878315335	0.0002071	0.004814	0.27828832
ENSG00000213347	MXD3	5.309948698	0.0002083	0.004833	-0.2959826
ENSG00000118971	CCND2	9.000796286	0.0002109	0.004886	-0.2253361
ENSG00000198753	PLXNB3	5.098054857	0.0002115	0.004891	0.35437547
ENSG00000243449	NICOL1	5.276189857	0.0002118	0.004891	-0.3139515
ENSG00000122705	CLTA	8.254598649	0.0002123	0.004894	-0.2314584
ENSG00000100889	PCK2	0.516064094	0.0002127	0.004897	1.46023833
ENSG00000110955	ATP5F1B	9.677898489	0.0002134	0.0049	-0.2290296
ENSG00000073060	SCARB1	7.437408473	0.0002135	0.0049	-0.230464
ENSG00000165323	FAT3	7.405768538	0.000216	0.004941	0.33208842
ENSG00000160014	CALM3	10.24703514	0.000216	0.004941	-0.228352
ENSG00000023909	GCLM	8.208481334	0.0002182	0.004973	-0.2254821
ENSG00000064961	HMG20B	6.951322274	0.0002182	0.004973	-0.2404582
ENSG00000222009	BTBD19	3.058224564	0.0002184	0.004973	0.61515315
ENSG00000080819	CPOX	4.013497022	0.0002191	0.00498	-0.4412529
ENSG00000106333	PCOLCE	7.52967575	0.0002237	0.005078	-0.2213025
ENSG00000077713	SLC25A43	4.687954837	0.0002244	0.005086	-0.3478973
ENSG00000181396	OGFOD3	4.960112124	0.0002254	0.005096	-0.3391489
ENSG00000175334	BANF1	7.172340863	0.0002256	0.005096	-0.2309593
ENSG00000273117	INSIG1-DT	1.494872154	0.0002278	0.00513	-0.9933491
ENSG00000177889	UBE2N	7.418153103	0.0002278	0.00513	-0.2246207
ENSG00000269825	NA	3.522138817	0.0002295	0.005161	0.50252659

ENSG00000072062	PRKACA	7.872260539	0.0002332	0.005236	-0.2286078
ENSG00000104388	RAB2A	8.278521227	0.0002369	0.005311	-0.2125465
ENSG00000123700	KCNJ2	0.642159228	0.0002388	0.005345	1.50402883
ENSG00000173258	ZNF483	3.256338398	0.0002418	0.005405	0.56358553
ENSG00000131165	CHMP1A	7.390460978	0.0002446	0.005458	-0.2292291
ENSG00000182240	BACE2	4.243706101	0.0002452	0.005464	0.39553786
ENSG00000093000	NUP50	7.605714887	0.000248	0.005511	-0.2197754
ENSG00000065060	BLTP3A	5.60503788	0.0002481	0.005511	0.27263805
ENSG00000237515	SHISA9	7.246509366	0.0002498	0.005541	0.23415068
ENSG00000152457	DCLRE1C	4.599878393	0.0002503	0.005542	0.35597145
ENSG00000187109	NAP1L1	9.667368302	0.0002509	0.005548	-0.2161949
ENSG00000179604	CDC42EP4	6.248934371	0.0002516	0.005556	-0.2816626
ENSG00000253910	PCDHGB2	3.795553817	0.0002544	0.005609	0.46761927
ENSG00000157045	NTAN1	4.907521473	0.0002569	0.005654	-0.336672
ENSG00000228486	C2orf92	2.461209155	0.0002592	0.00569	0.69197719
ENSG00000138606	SHF	2.947035591	0.0002593	0.00569	0.59683298
ENSG00000107036	RIC1	5.971424078	0.0002596	0.00569	0.26234959
ENSG00000102780	DGKH	5.707674726	0.0002709	0.005928	0.30365889
ENSG00000114268	PFKFB4	3.068717706	0.0002714	0.005929	-0.5806207
ENSG00000258077	NA	2.543307387	0.0002718	0.005929	-0.6861087
ENSG00000189308	LIN54	5.724716975	0.0002725	0.005936	0.28552979
ENSG00000186141	POLR3C	4.367889874	0.0002732	0.005938	0.38479579
ENSG00000008018	PSMB1	7.30292873	0.0002739	0.005938	-0.2228279
ENSG00000291068	NA	2.502459321	0.0002739	0.005938	0.6984334
ENSG00000061656	SPAG4	2.257635196	0.0002743	0.005938	0.72736223
ENSG00000137073	UBAP2	6.393293759	0.000275	0.005938	0.24294052
ENSG00000119318	RAD23B	8.153379068	0.000275	0.005938	-0.2095181
ENSG00000231889	TRAF3IP2-AS1	2.472968143	0.0002781	0.005985	0.69433183
ENSG00000287275	NA	-1.205973943	0.0002783	0.005985	3.1594743
ENSG00000078237	TIGAR	4.080102926	0.0002784	0.005985	0.41383123
ENSG00000180340	FZD2	5.130578672	0.0002795	0.005993	-0.315538
ENSG00000183696	UPP1	7.995125475	0.0002796	0.005993	-0.2148533
ENSG00000169764	UGP2	6.789509692	0.0002812	0.006017	-0.2267698
ENSG00000167978	SRRM2	9.653348304	0.000285	0.00609	0.26156204
ENSG00000162241	SLC25A45	3.30041254	0.0002854	0.00609	0.56596491
ENSG00000078699	CBFA2T2	5.61861697	0.0002858	0.006091	0.27151089
ENSG00000151640	DPYSL4	7.002312415	0.0002867	0.0061	0.22391172
ENSG00000007520	TSR3	6.231530365	0.0002897	0.00615	-0.2566773
ENSG00000266714	MYO15B	4.861259057	0.0002899	0.00615	0.48681608
ENSG00000173726	TOMM20	8.312474819	0.000291	0.006163	-0.2082023

ENSG00000146729	NIPSNAP2	6.4160115	0.0002913	0.006163	-0.2423704
ENSG00000120942	UBIAD1	6.000124691	0.0002921	0.006171	-0.2586226
ENSG00000125170	DOK4	6.927253344	0.0002946	0.006214	-0.2233892
ENSG00000149182	ARFGAP2	6.927555609	0.0002966	0.006246	-0.2214569
ENSG00000107949	BCCIP	6.08441817	0.000297	0.006246	-0.2483062
ENSG00000163584	RPL22L1	6.442568371	0.0002989	0.006272	-0.2545434
ENSG00000196072	BLOC1S2	5.789564861	0.0002991	0.006272	0.27565396
ENSG00000125354	SEPTIN6	6.446179786	0.0003014	0.006312	-0.2424749
ENSG00000181291	TMEM132E	8.796894163	0.0003038	0.006341	-0.2197007
ENSG00000108953	YWHAE	9.375171235	0.0003041	0.006341	-0.2243124
ENSG00000135940	COX5B	6.381979375	0.0003041	0.006341	-0.2593646
ENSG00000083312	TNPO1	8.033042583	0.0003077	0.006399	0.20931144
ENSG00000170579	DLGAP1	6.651709996	0.0003077	0.006399	-0.2310363
ENSG00000205476	CCDC85C	7.597654086	0.0003092	0.00642	-0.2269598
ENSG00000188986	NELFB	8.881584824	0.0003121	0.006471	-0.2383425
ENSG00000109519	GRPEL1	5.557838906	0.0003142	0.006504	-0.2841927
ENSG00000203499	IQANK1	2.691389619	0.0003182	0.006579	0.65951499
ENSG00000122545	SEPTIN7	8.171435088	0.0003214	0.006627	-0.2062208
ENSG00000167371	PRRT2	2.111571512	0.0003217	0.006627	0.77465977
ENSG00000136040	PLXNC1	5.38989032	0.000323	0.006627	0.28462952
ENSG00000272391	POM121C	7.144730836	0.0003231	0.006627	0.22397745
ENSG00000181163	NPM1	9.611037075	0.0003234	0.006627	-0.2150682
ENSG00000103710	RASL12	-0.46865128	0.0003236	0.006627	-2.059661
ENSG00000123975	CKS2	7.48826353	0.0003237	0.006627	-0.2405005
ENSG00000182534	MXRA7	7.103623468	0.0003249	0.006643	-0.2458106
ENSG00000258890	CEP95	5.003471239	0.0003256	0.006647	0.40377271
ENSG00000131196	NFATC1	3.480435165	0.0003306	0.00674	-0.4977504
ENSG00000111667	USP5	7.402966498	0.0003311	0.006741	-0.218239
ENSG00000196876	SCN8A	3.798085192	0.0003341	0.006792	0.45273249
ENSG00000163602	RYBP	5.666092402	0.000335	0.006801	0.27152851
ENSG00000105568	PPP2R1A	8.711025842	0.0003358	0.006807	-0.220166
ENSG00000125812	GZF1	5.367838298	0.0003386	0.006854	0.29560149
ENSG00000115163	CENPA	3.964631666	0.0003395	0.006859	-0.4377539
ENSG00000110719	TCIRG1	6.555613088	0.0003397	0.006859	0.23520236
ENSG00000189403	HMGB1	9.536757391	0.0003405	0.006865	-0.2198076
ENSG00000100380	ST13	8.169498489	0.0003426	0.006896	-0.2132217
ENSG00000030419	IKZF2	3.490127655	0.000343	0.006896	0.50328138
ENSG00000108671	PSMD11	6.836425512	0.0003558	0.007141	-0.2225477
ENSG00000213398	LCAT	4.240535566	0.0003564	0.007141	0.47180704
ENSG00000182481	KPNA2	7.876284007	0.0003571	0.007141	-0.2131075
ENSG00000152642	GPD1L	7.219031403	0.0003581	0.007141	-0.2377499

ENSG00000198168	SVIP	7.154802229	0.0003585	0.007141	-0.2166168
ENSG00000137312	FLOT1	7.776891622	0.0003586	0.007141	-0.208278
ENSG00000167191	GPRC5B	7.622726515	0.0003588	0.007141	-0.2092118
ENSG00000124562	SNRPC	6.377552847	0.0003596	0.007141	-0.2443116
ENSG00000168763	CNNM3	5.461775833	0.0003597	0.007141	0.27689481
ENSG00000103540	CCP110	5.252353474	0.00036	0.007141	0.31441974
ENSG00000147324	MFHAS1	6.518441316	0.0003608	0.007145	0.24260616
ENSG00000183684	ALYREF	7.506264755	0.0003708	0.007335	-0.2197876
ENSG00000132819	RBM38	5.218638314	0.000376	0.007424	0.30578473
ENSG00000187678	SPRY4	8.40725757	0.0003765	0.007424	0.21529683
ENSG00000121957	GPSM2	5.348938658	0.0003769	0.007424	-0.2818009
ENSG00000142669	SH3BGRL3	6.056110382	0.0003803	0.007475	-0.2681065
ENSG00000171428	NAT1	0.769678909	0.0003805	0.007475	1.20458519
ENSG00000169871	TRIM56	6.435598319	0.0003819	0.007493	0.2341949
ENSG00000092199	HNRNPC	9.443928953	0.0003901	0.007643	-0.2117355
ENSG00000099840	IZUMO4	1.668867021	0.0003979	0.007785	0.8846806
ENSG00000198824	CHAMP1	6.108713539	0.000401	0.007836	0.24791332
ENSG00000130202	NECTIN2	9.19072143	0.0004038	0.00788	-0.221421
ENSG00000189190	ZNF600	2.415483375	0.0004049	0.00789	0.69847931
ENSG00000157020	SEC13	7.543701879	0.0004055	0.007893	-0.2181957
ENSG00000186193	SAPCD2	8.780429011	0.0004089	0.007947	-0.2043952
ENSG00000088305	DNMT3B	6.904856783	0.0004241	0.008232	-0.2205091
ENSG00000122068	FYTTD1	6.716097843	0.0004272	0.008263	-0.2364519
ENSG00000125817	CENPB	8.201771494	0.0004274	0.008263	-0.2219734
ENSG00000114423	CBLB	6.027056153	0.0004274	0.008263	0.25098451
ENSG00000117593	DARS2	5.825666162	0.0004285	0.008267	-0.2540972
ENSG00000168386	FILIP1L	0.172484607	0.0004287	0.008267	1.5074748
ENSG00000115183	TANC1	7.017502564	0.0004314	0.008308	0.21490189
ENSG00000158457	TSPAN33	-0.412481946	0.0004323	0.008314	1.91552172
ENSG00000129315	CCNT1	6.183475559	0.000437	0.008374	0.23962885
ENSG00000103194	USP10	7.519146689	0.0004373	0.008374	-0.2165569
ENSG00000185133	INPP5J	1.847104612	0.0004374	0.008374	0.81204187
ENSG00000066855	MTFR1	5.479665257	0.0004377	0.008374	-0.2733958
ENSG00000198954	KIFBP	6.589094155	0.0004401	0.00841	-0.2245529
ENSG00000278864	NA	3.047186827	0.0004408	0.008412	0.61469164
ENSG00000185100	ADSS1	2.740152491	0.0004424	0.008418	0.62702362
ENSG00000179010	MRFAP1	8.987747673	0.0004427	0.008418	-0.2073451
ENSG00000099250	NRP1	4.933576164	0.0004428	0.008418	0.31163386
ENSG00000102908	NFAT5	4.951216733	0.000446	0.008467	0.39637433
ENSG00000180198	RCC1	6.101747064	0.0004501	0.008532	-0.2440041
ENSG00000103264	FBXO31	5.749968892	0.0004508	0.008535	-0.2529568

ENSG00000162522	NHSL3	4.054482118	0.0004523	0.008553	0.40038522
ENSG00000114686	MRPL3	5.885808571	0.0004553	0.008599	-0.2717622
ENSG00000123159	GIPC1	7.144513704	0.0004633	0.008739	-0.2695824
ENSG00000118680	MYL12B	8.005671862	0.0004667	0.008788	-0.2231315
ENSG00000136518	ACTL6A	6.173361394	0.0004671	0.008788	-0.2388247
ENSG00000105771	SMG9	6.664359755	0.0004708	0.008845	0.24369853
ENSG00000148019	CEP78	6.08288405	0.0004725	0.008866	0.24792937
ENSG00000146555	SDK1	5.502218648	0.0004752	0.008905	0.2860012
ENSG00000189343	RPS2P46	4.899262518	0.0004767	0.00892	-0.3331276
ENSG00000240342	RPS2P5	8.562882926	0.0004772	0.00892	-0.2193906
ENSG00000188976	NOC2L	6.592534993	0.00048	0.008961	-0.2393638
ENSG00000138777	PPA2	5.854147693	0.0004815	0.008977	-0.2544645
ENSG00000123472	ATPAF1	6.272441184	0.0004828	0.00899	-0.2381616
ENSG00000100294	MCAT	4.368395017	0.0004837	0.008995	-0.3819376
ENSG00000182154	MRPL41	7.650948739	0.0004867	0.009039	-0.2094811
ENSG00000128016	ZFP36	2.796239588	0.0004878	0.009049	0.6309468
ENSG00000087586	AURKA	6.085133963	0.0004937	0.009145	-0.23818
ENSG00000223959	AFG3L1P	4.659398225	0.0004964	0.009184	0.36226168
ENSG00000105953	OGDH	6.362557515	0.000497	0.009184	-0.2437913
ENSG00000148339	SLC25A25	5.801552747	0.0005024	0.009253	0.25023927
ENSG00000095637	SORBS1	2.647203794	0.0005026	0.009253	0.68857786
ENSG00000135842	NIBAN1	4.265715494	0.0005027	0.009253	0.38805867
ENSG00000125656	CLPP	6.968690854	0.0005032	0.009253	-0.2207741
ENSG00000233016	SNHG7	7.803259208	0.0005052	0.009276	-0.2179262
ENSG00000177600	RPLP2	9.696676397	0.0005058	0.009276	-0.2067103
ENSG00000182628	SKA2	7.168049938	0.0005086	0.009315	-0.2102916
ENSG00000275993	NA	4.52350658	0.0005098	0.009326	-0.343623
ENSG00000165672	PRDX3	7.558313887	0.0005108	0.009333	-0.2026957
ENSG00000183479	TREX2	0.963289179	0.0005145	0.009389	1.14699948
ENSG00000068650	ATP11A	6.784232784	0.0005164	0.009412	0.24354459
ENSG00000154358	OBSCN	6.44067674	0.000518	0.009429	0.24231378
ENSG00000175792	RUVBL1	6.418595888	0.00052	0.009454	-0.2414261
ENSG00000115053	NCL	9.850663398	0.0005267	0.009555	-0.214172
ENSG00000112763	BTN2A1	4.073327792	0.0005269	0.009555	0.4107018
ENSG00000180626	ZNF594	3.903398424	0.0005288	0.009578	0.43682693
ENSG00000092841	MYL6	8.497216362	0.0005308	0.009593	-0.2003204
ENSG00000112659	CUL9	5.335567714	0.000531	0.009593	0.27912355
ENSG00000164877	MICALL2	5.284977695	0.000532	0.0096	0.32927961
ENSG00000110422	HIPK3	7.581299906	0.0005353	0.009648	0.21699381
ENSG00000125648	SLC25A23	7.306937545	0.0005433	0.00978	-0.2126829
ENSG00000145012	LPP	6.284890378	0.0005483	0.009847	0.29697137

ENSG00000160991	ORAI2	6.539208371	0.0005486	0.009847	-0.2212629
ENSG00000168944	CEP120	5.356796454	0.000549	0.009847	0.27240136
ENSG00000103995	CEP152	5.594979304	0.0005573	0.009984	0.33065193
