# Supplementary material for: The CIC::DUX4 oncoprotein maintains DNA integrity through direct regulation of the catalytic subunit of DNA polymerase epsilon (POLE)
Source: Oncogene. 2025 Aug 4;44(38):3598–608. doi: 10.1038/s41388-025-03507-9 (PMC12436161; doi:10.1038/s41388-025-03507-9)
Supplement: Supplementary file 2 — Supplementary Figures [file 41388_2025_3507_MOESM2_ESM.pdf]

Supplementary data:

The CIC::DUX4 oncoprotein maintains DNA integrity through direct regulation of the catalytic subunit of DNA polymerase epsilon (POLE).

Authors: Zeinab Kosibaty, Cuyler Luck, and Ross A. Okimoto

The supplementary data file contains 13 figures.

A

Plasmid	PG4.10-POLE	PG4.10-mut-C7	PG4.10-mut-B1	PG4.10-mut-C4
Length bp	1640	1240	588	260
TGAATGAG	22	17	6	2
TGAATGAA	1	0	1	0

B

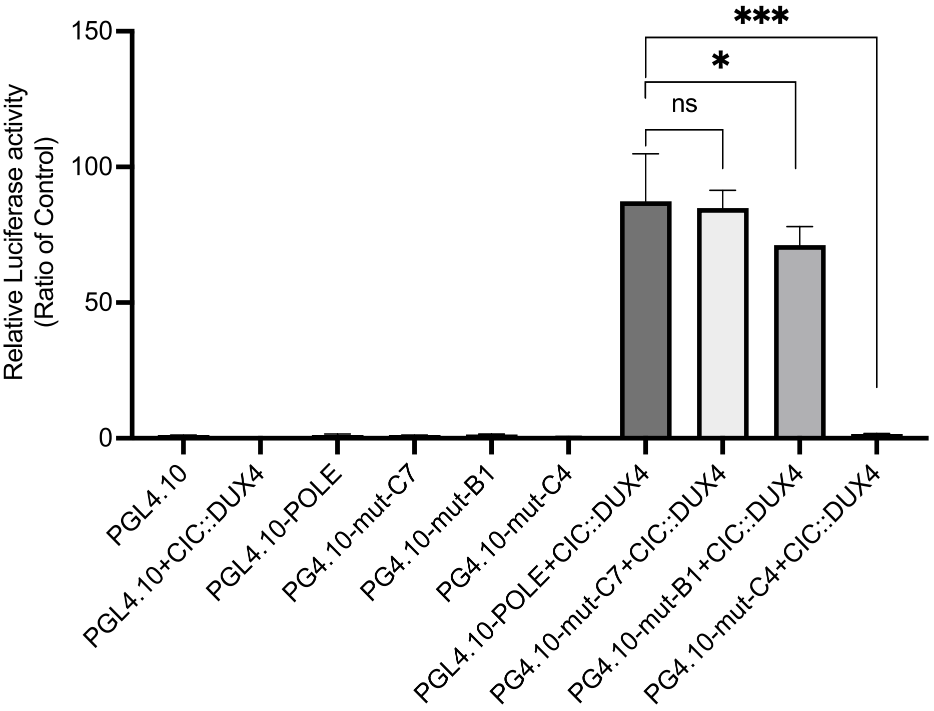

Supplementary Figure 1. CIC binds and regulates *POLE* expression. (A) Table representation of luciferase reporter constructs. PGL4.10-POLE contains a ~1640 bp genomic sequence of the *POLE* promoter region that includes one canonical CIC::DUX4 binding motif (TGAATGAA) and 22 variant motifs (TGAATGAG). Three mutant constructs were generated: PGL4.10-mut-C7 (~1240 bp), containing 17 TGAATGAG motifs, PGL4.10-mut-B1 (~588 bp), containing six TGAATGAG motifs and one TGAATGAA motif, and PGL4.10-mut-C4 (~260 bp) containing two TGAATGAG motifs. (B) Relative luciferase activity in HEK293T cells transfected with various constructs: PGL4.10, PGL4.10-POLE, PGL4.10+CIC::DUX4, PGL4.10-POLE, PGL4.10-mut-C7, PGL4.10-mut-B1, PGL4.10-mut-C4, PGL4.10-POLE+CIC::DUX4, PGL4.10-mut-C7+CIC::DUX4, PGL4.10-mut-B1+CIC::DUX4, and PGL4.10-mut-C4+CIC::DUX4. Error bars represent standard deviation (SD). Statistical significance was assessed using Student's t-test: $^{*}P < 0.05$, $^{***}P < 0.001$. Data shown are representative of two independent experiments.

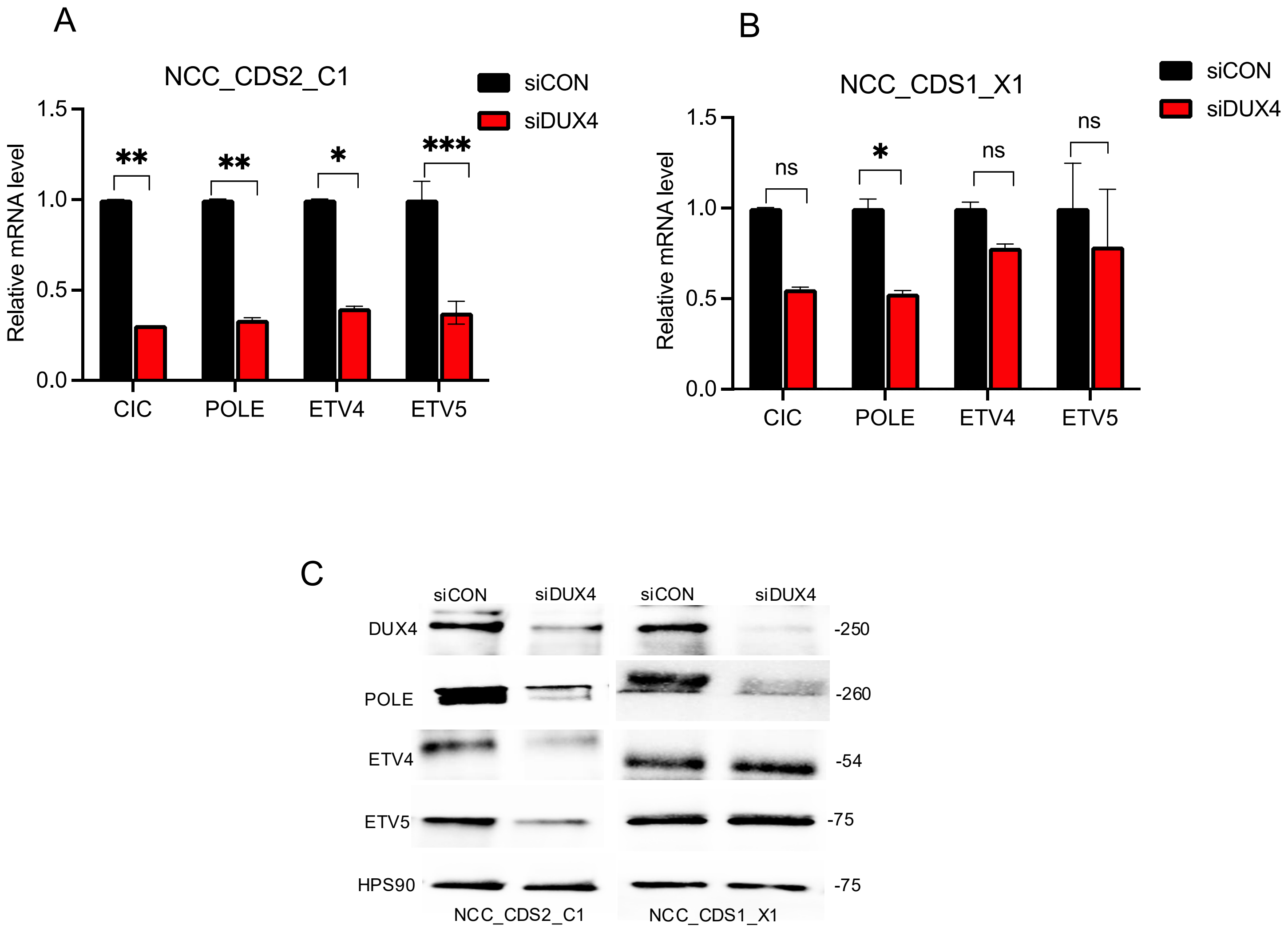

Supplementary Figure 2. Impact of DUX4 supression on POLE and ETV4/5 expression in CIC::DUX4 sarcoma cells. (A, B) Relative mRNA expression levels of *CIC*, *POLE*, *ETV4,* and *ETV5* in NCC_CDS2_C1 and NCC_CDS1_X1_C1 cells transfected with *siDUX4* (CIC::DUX4 knockdown) or scramble control (siCON). (C) Immunoblots of DUX4, POLE and ETV4, and ETV5 from NCC_CDS2_C1 and NCC_CDS1_X1_C1 cells transfected with *siDUX4* or siCON for 72 hours. Error bars represent standard deviation (SD); statistical significance was assessed Student's t-test. **P < .05, **P < .01, ***P < .001*. Data represent results from two independent experiments.

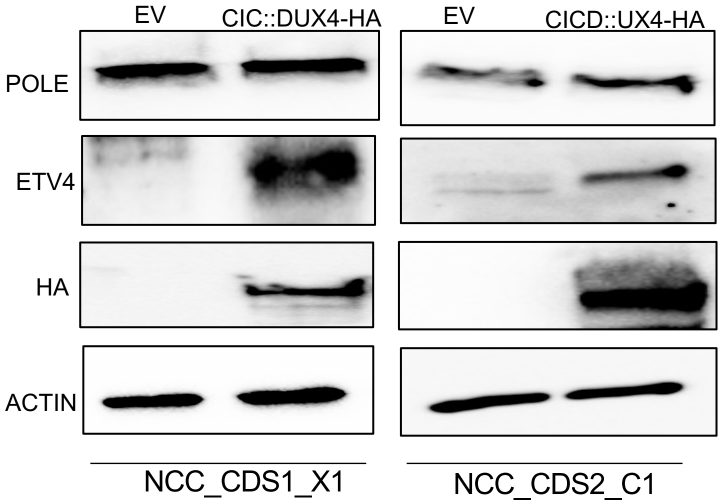

Supplementary Figure 3. Ectopic expression of CIC::DUX4 in NCC-CDS1-X1-C1 and NCC-CDS2-C1 cells.

Immunoblot analysis of NCC_CDS1_X1_C1 and NCC_CDS2-C1 cells transfected with CIC::DUX4-HA or empty vector (EV) control. POLE expression levels showed a slight increase in NCC_CDS2_C1 cells post-transfection with CIC::DUX4-HA, while no change was observed in NCC_CDS1_X1_C1 cells. ETV4 expression was markedly elevated in both cell lines following CIC::DUX4-HA transfection compared to EV. ACTIN was used as a loading control. Data represent results from three independent experiments.

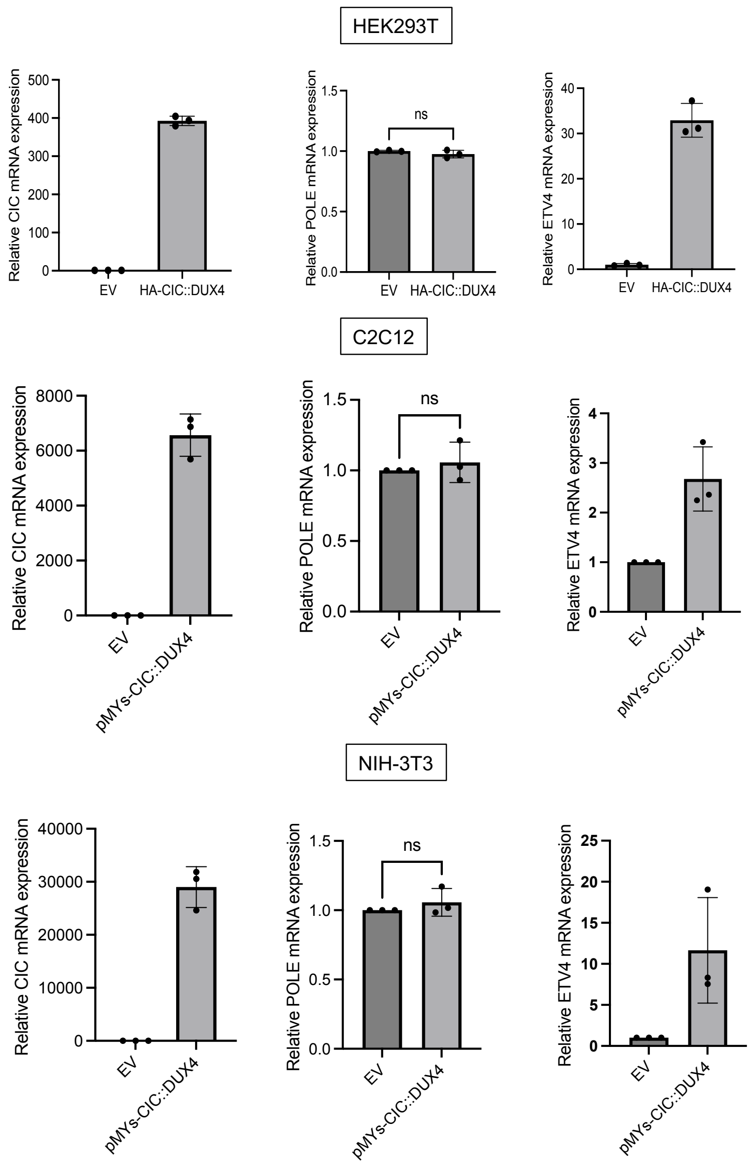

Supplementary Figure 4. **Ectopic expression of CIC::DUX4 does not affect POLE in non-sarcoma cell lines (HEK293T, C2C12, and NIH-3T3).**

Relative mRNA expression levels of *CIC, POLE,* and *ETV4,* were measured in HEK293T (human embryonic kidney), C2C12 (myoblasts), and NIH-3T3 (fibroblasts) cells transfected with human ectopic CIC::DUX4-HA, PMYS-CIC::DUX4, or an empty vector (EV) control. Gene expression levels were normalized to GAPDH, and fold change was calculated relative to the EV control. Error bars represent the standard deviation (SD) of technical replicates (n=3). Statistical analysis was performed with Student's t-test, with $P > 0.05$ considered non-significant (ns).

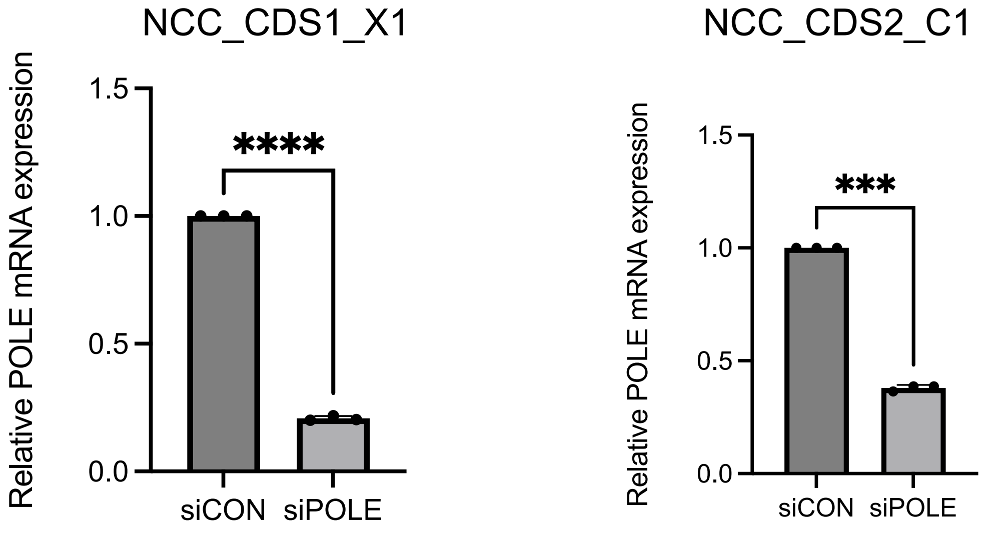

Supplementary Figure 5. Validation of POLE mRNA silencing in CIC::DUX4 sarcoma cells. RT-PCR analysis confirmed the suppression of POLE mRNA expression levels in the NCC_CDS1_X1_C1 and NCC_CDS2_C1 sarcoma cell lines 48 hours post-transfection. Cells were transfected with siRNAs targeting *POLE* or a scrambled control (siCON). mRNA levels were normalized to GAPDH as an internal control.

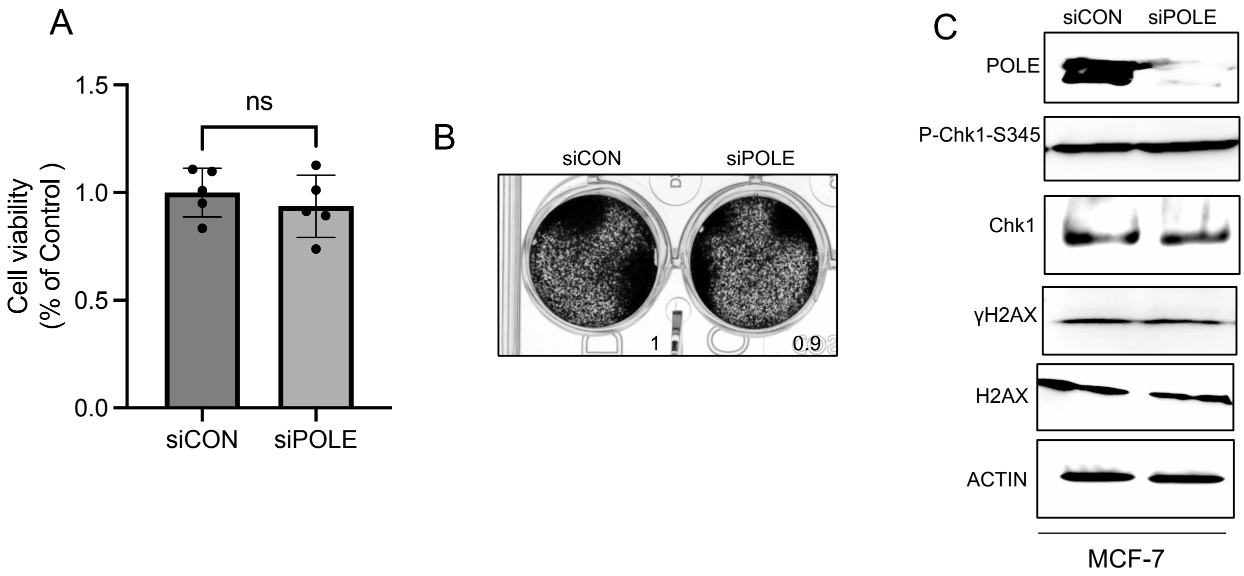

Supplementary Figure 6. Impact of POLE suppression on DNA damage and viability of MCF7 cells. (A) Relative cell viability assays (CellTiter-Glo) comparing MCF7 cells expressing *siPOLE* with siCON. (B) Crystal violet assay in MCF7 cells with *siPOLE* versus siCON expression. (C) Immunoblot analysis of POLE, p-Chk1 (Serine 345), total Chk1, γH2AX, and total H2AX in MCF7 cells expressing *siPOLE* or siCON. ACTIN serves as the loading control.

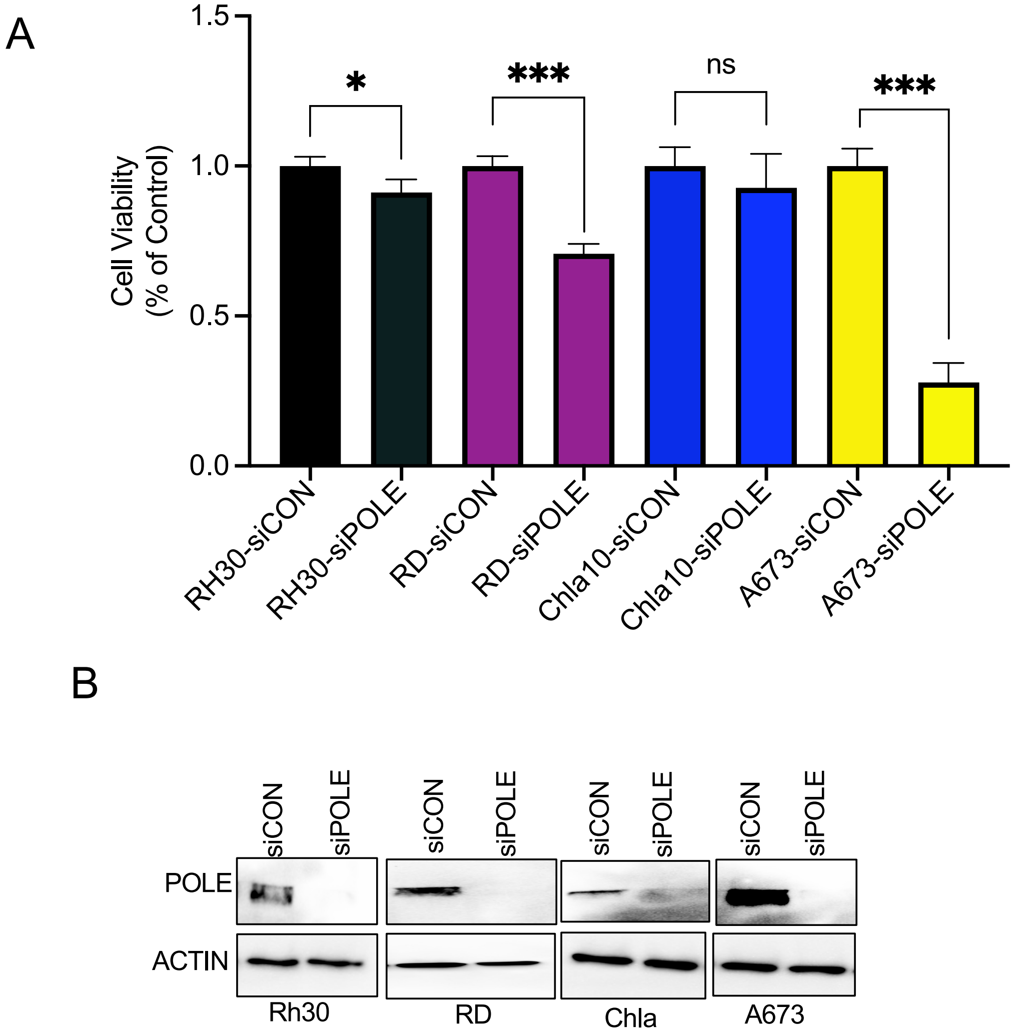

Supplementary Figure 7. Impact of POLE suppression on sarcoma cells.

(A) Relative cell viability was assessed across a panel of sarcoma subtypes, including rhabdomyosarcoma (Rh30, RD) and Ewing sarcoma (CHLA10, A673). Cells were transfected with *siPOLE* or scrambled control (siCON) for 48 hours. (B) Immunoblot analysis of POLE expression in the panel of sarcoma subtypes was performed following transfection with *siPOLE* or siCON. Error bars represent standard deviation (SD); Data represent results from at least three independent experiments. **P < .05, ***P < .001.*

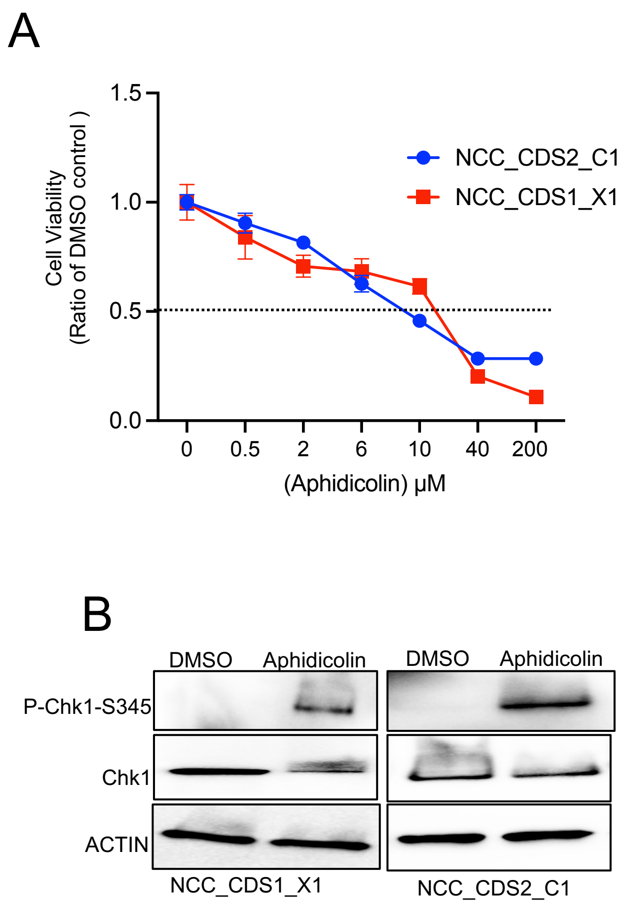

Supplementary Figure 8. Therapeutic impact of Aphidicolin on CIC::DUX4 sarcoma cells.

(A) Relative cell viability was assessed in CIC::DUX4 sarcoma cells treated with serial concentrations of Aphidicolin for 48 hours. Error bars represent standard deviation (SD), and data are derived from at least three independent experiments. (B) Immunoblot analysis demonstrating the levels of DNA damage markers, including phosphorylated CHK1, in NCC_CDS1_X1_C1 and NCC_CDS2_C1 cells treated with the IC50 dose of Aphidicolin compared to DMSO control. Representative results from duplicate experiments are shown.

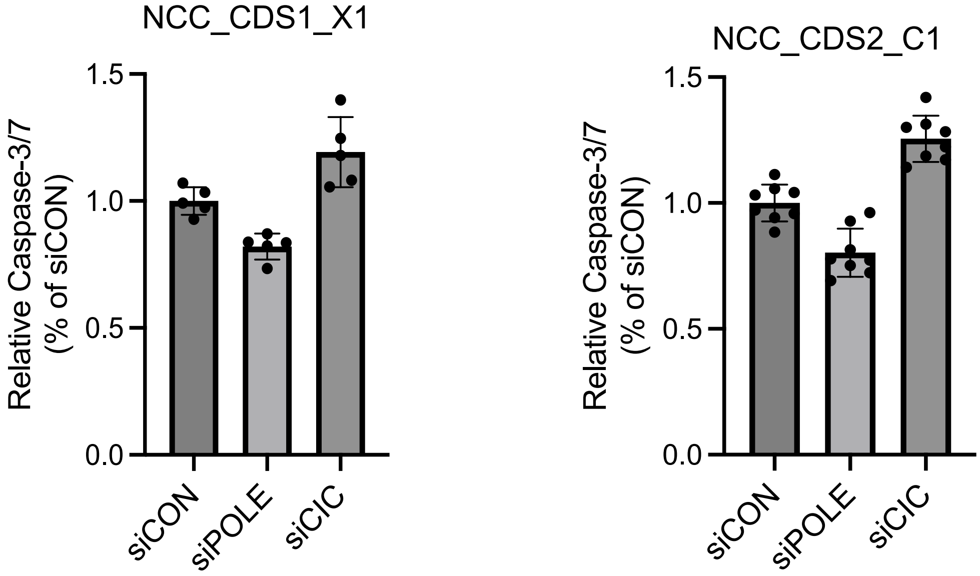

Supplementary Figure 9. Evaluation of caspase activity in response to POLE and CIC::DUX4 silencing in CIC::DUX4 sarcoma cells. Caspase 3/7 activity was assessed in NCC_CDS1_X1_C1 and NCC_CDS2_C1 cells following silencing of POLE or CIC::DUX4. Error bars represent standard deviation (SD).

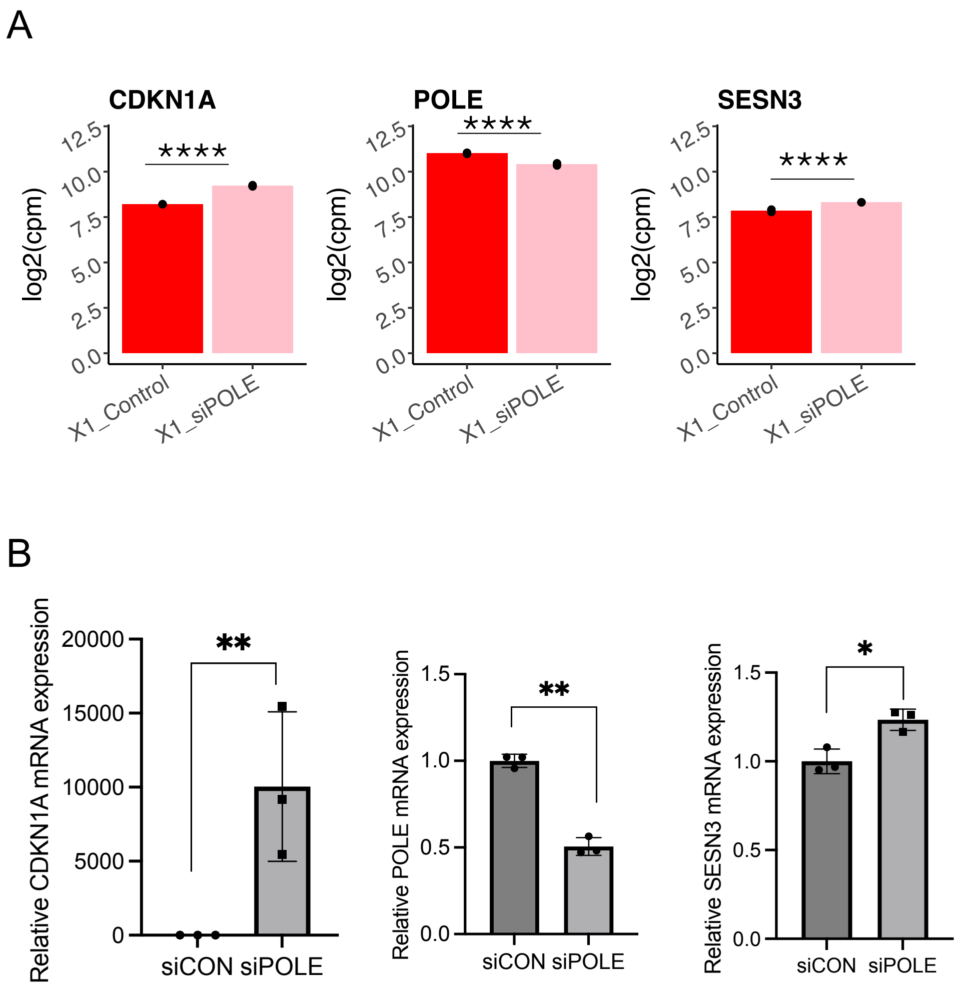

Supplementary Figure 10. Expression of mRNA XDKN1A, POLE, SESN3. (A) Log2(counts per million) measurements for CDKN1A, POLE, and SESN3 genes in NCC_CDS1_X1_C1. Bars represent mean values. In the edgeR differential expression analysis using quasi-likelihood F tests. all three genes were significantly different for siCON vs *siPOLE*. The FDR-adjusted p-values are: ****p < 0.0001. (B) RT-PCR analysis showed the suppression of CDKN1A, POLE, SESN3 mRNA expression levels in the NCC_CDS2_C1 sarcoma cells 48 hours post-transfection. Cells were transfected with siRNAs targeting *POLE* or a scrambled control (siCON). mRNA levels were normalized to GAPDH as an internal control. The RT-PCR data represent results from at least three independent experiments. **P < .05, ***P < .001.*

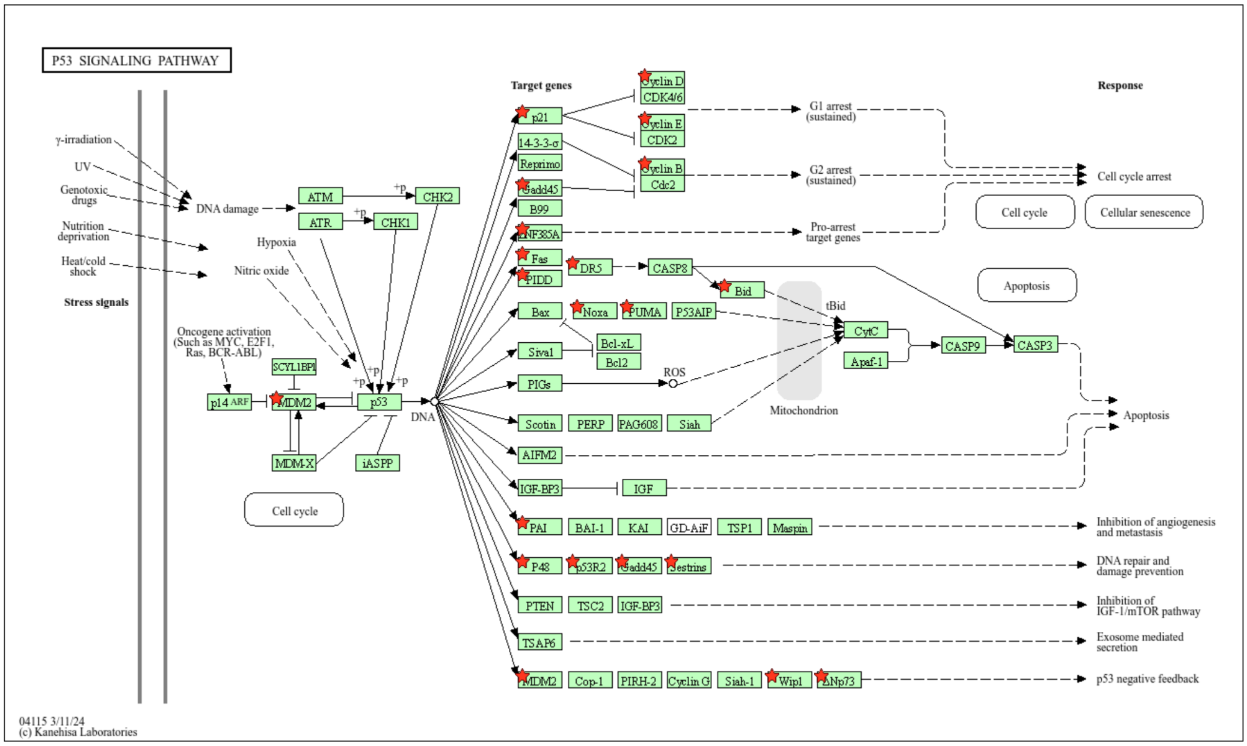

Supplementary Figure 11. Visualization of the p53 signaling pathway derived from KEGG analysis of NCC_CDS1_X1_C1 in POLE-suppressed versus control cells. Genes highlighted with red stars indicate putative targets that are regulated by POLE.

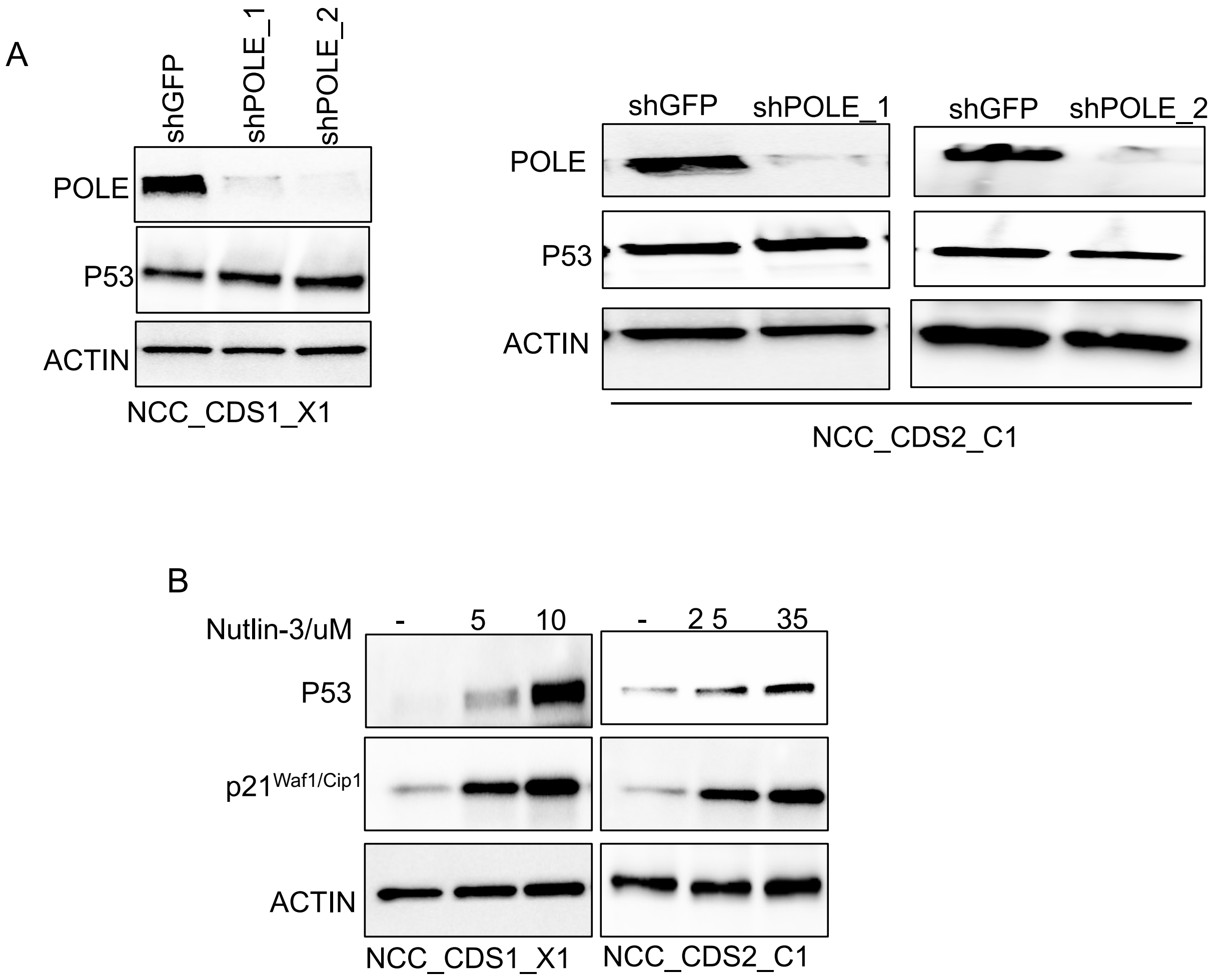

Supplementary Figure 12. Activation of the p53-p21 pathway independent of POLE in CIC::DUX4 sarcoma cells. (A) Western blot analysis was conducted to assess the expression levels of p53 in NCC_CDS1_X1_C1 and NCC_CDS2_C1 cells expressing shGFP, *shPOLE_1*, or *shPOLE_2*. (B) Western blot analysis of p21 expression in NCC_CDS1_X1_C1 cells treated with Nutlin-3 at concentrations of 5 μM and 10 μM, and in NCC_CDS2_C1 cells treated at concentrations of 25 μM and 30 μM, with DMSO serving as the control.

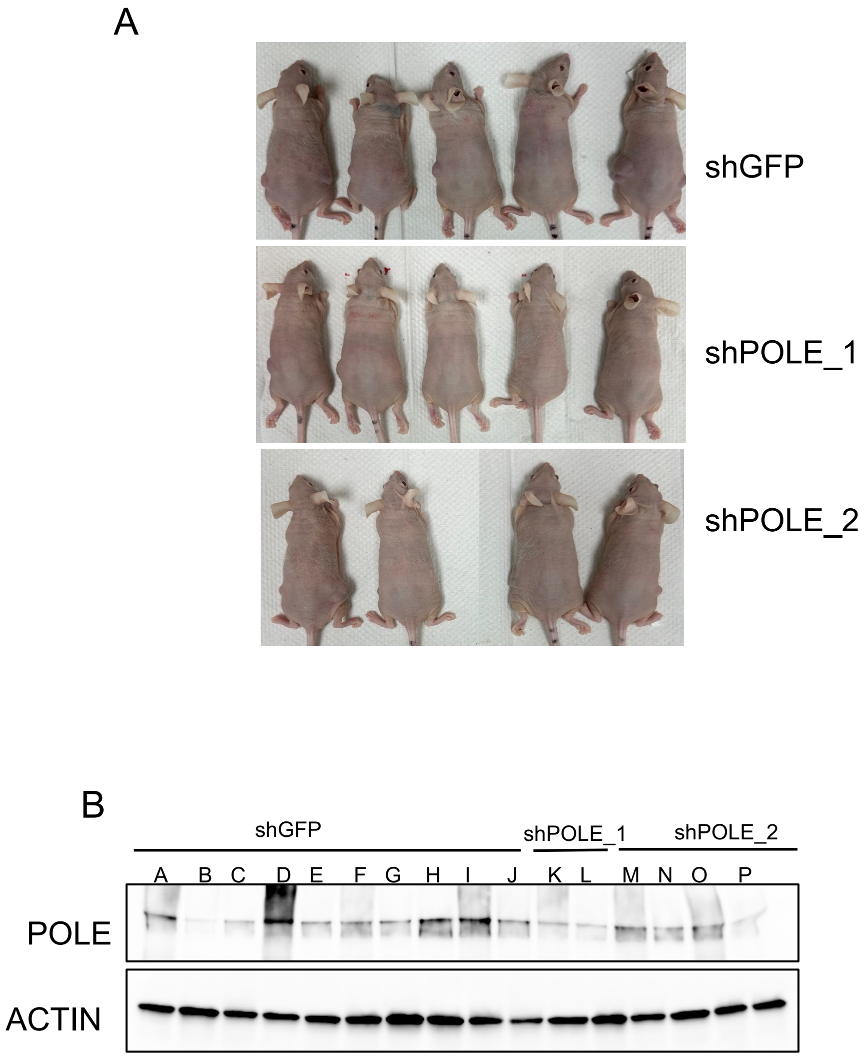

Supplementary Figure 13. (A) Representative figures illustrate the subcutaneous injection of NCC_CDS2_C1 cells expressing shGFP, *shPOLE_1*, or *shPOLE_2* into immunodeficient nude mice. Tumors were formed in (9/10) mice injected with shGFP, *shPOLE_1* (2/10), and *shPOLE_2* (4/8). (B) Immunoblot analysis demonstrates the expression of POLE in tumor explants from the shGFP (A-J) *shPOLE_1* (K-L) and *shPOLE_2* (M-P) groups. ACTIN serves as the loading control
